# Mechanistic insights into polyphenolic constituents from traditional Chinese medicines in inhibiting colorectal cancer metastasis

**DOI:** 10.1007/s13659-026-00622-2

**Published:** 2026-05-08

**Authors:** Ming-Hong Li, Lu Liu

**Affiliations:** Yunnan Yunzhong Institute of Nutrition and Health, College of Traditional Chinese Medicine, Yunnan University of Chinese Medicine, Kunming, 650500 People’s Republic of China

**Keywords:** Colorectal cancer, Metastasis, TCM, Polyphenolic

## Abstract

Colorectal cancer (CRC) is the third most predominant cancer caused by genetic, environmental and nutritional factors and is the world’s second most significant cause of cancer-related mortality. During the process of tumor metastasis, firstly, cancer cell increases its proliferative capacity by reducing autophagy and apoptosis, and then the cancer cell capacity is stimulated by increasing the ability of tumors to absorb nutrients from the outside through angiogenesis. Both of the two steps can increase tumor migration and invasion. Finally, the purpose of tumor metastasis is achieved. By inducing autophagy and apoptosis of tumor cells, blocking the angiogenesis and inhibiting EMT outside the tumor can reduce the invasion and migration of cancer, and consequently achieve the purpose of inhibiting tumor metastasis. Traditional Chinese Medicine (TCM) has the characteristics of multiple targets, slight side effects and good therapeutic effects. Research of the anticancer effect of Polyphenolic in TCM has made considerable progress in recent years. This review explores the research achievements of Polyphenolic in TCM on CRC metastasis in the past ten years, summarizes the development direction of Polyphenolic in TCM on CRC metastasis research in the past ten years and makes a prospect for the future.

**Graphical Abstract:**

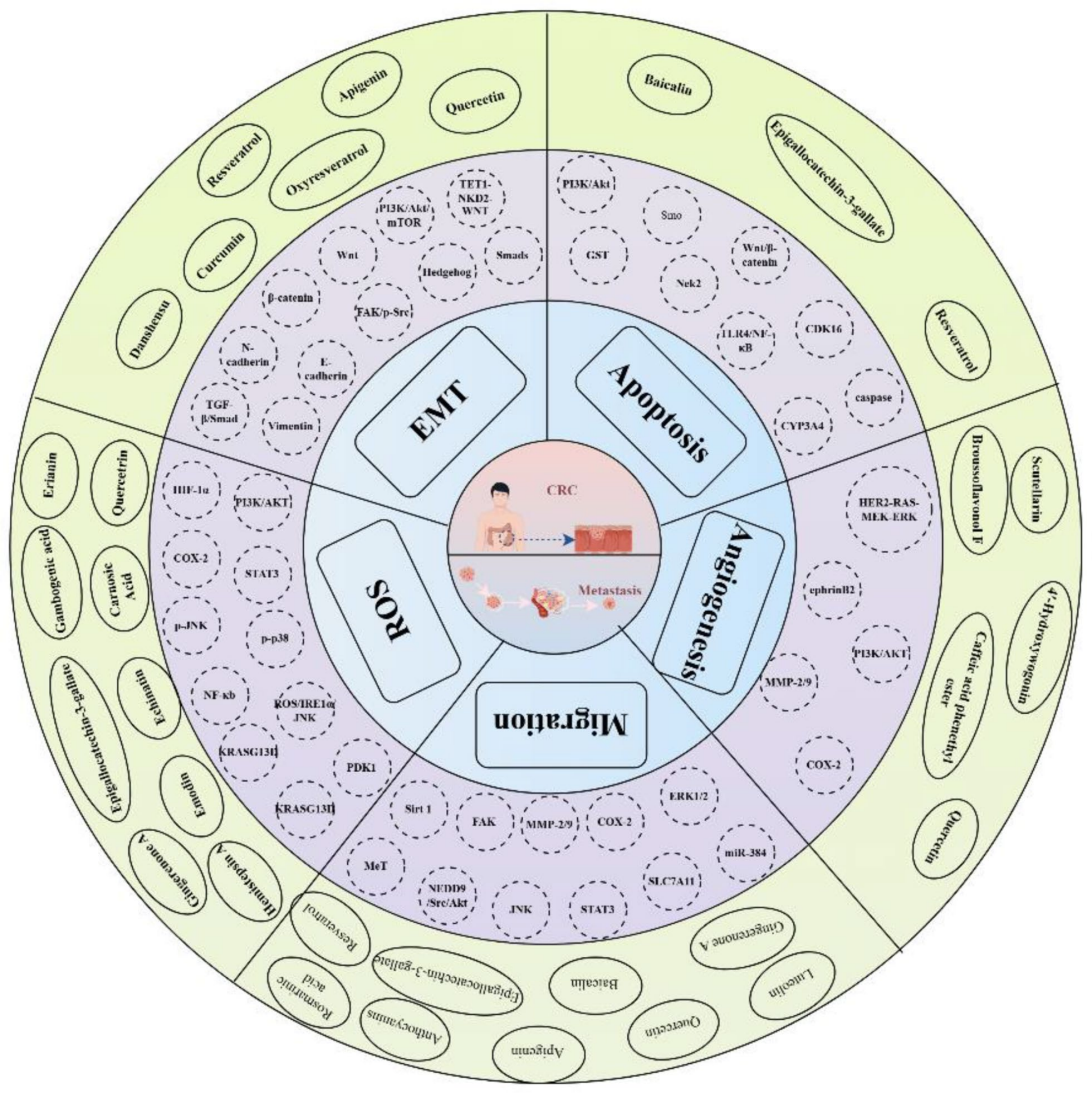

## Introduction

CRC is one of the most dangerous malignancies, spreading to the liver, lungs, ovaries, and other parts of the gastrointestinal system [1]. According to GLOBOCAN 2022 data, there were approximately 19.9 million new cancer cases and 9.7 million cancer-related deaths worldwide, with CRC accounting for around 1.14 million (5.7%) of the new cases and 0.54 million (5.5%) of the deaths. These statistics underscore a significant projected increase in cancer diagnoses, with the global incidence of CRC expected to approximately quadruple by 2050. Paralleled with the advances in surgical and allied specialties, the increasing number of effective drugs for colorectal cancer has led to substantial improvement of overall survival [2], the 5-year relative survival remains poor at only 65% [3]. The odds are worse for patients with metastatic disease as their 5-year survival rate is less than 20% [4], and 90% of cancer deaths are due to metastasis [5, 6]. Cancer metastasis represents the process of malignant tumor cells leaving the primary lesion, penetrating through the vessels and entering the host’s blood circulation and lymphatic circulation, or directly spreading through the body cavity, reaching the distant site and continuing to proliferate and grow, eventually forming the same type of tumor as the primary lesion [7]. Tumor metastasis is an important indicator of the development of malignancy [8]. Inhibiting tumor metastasis can inhibit tumor cells from metastasizing to other parts of the body, and thus delay tumor deterioration [9].

**Fig. 1:**
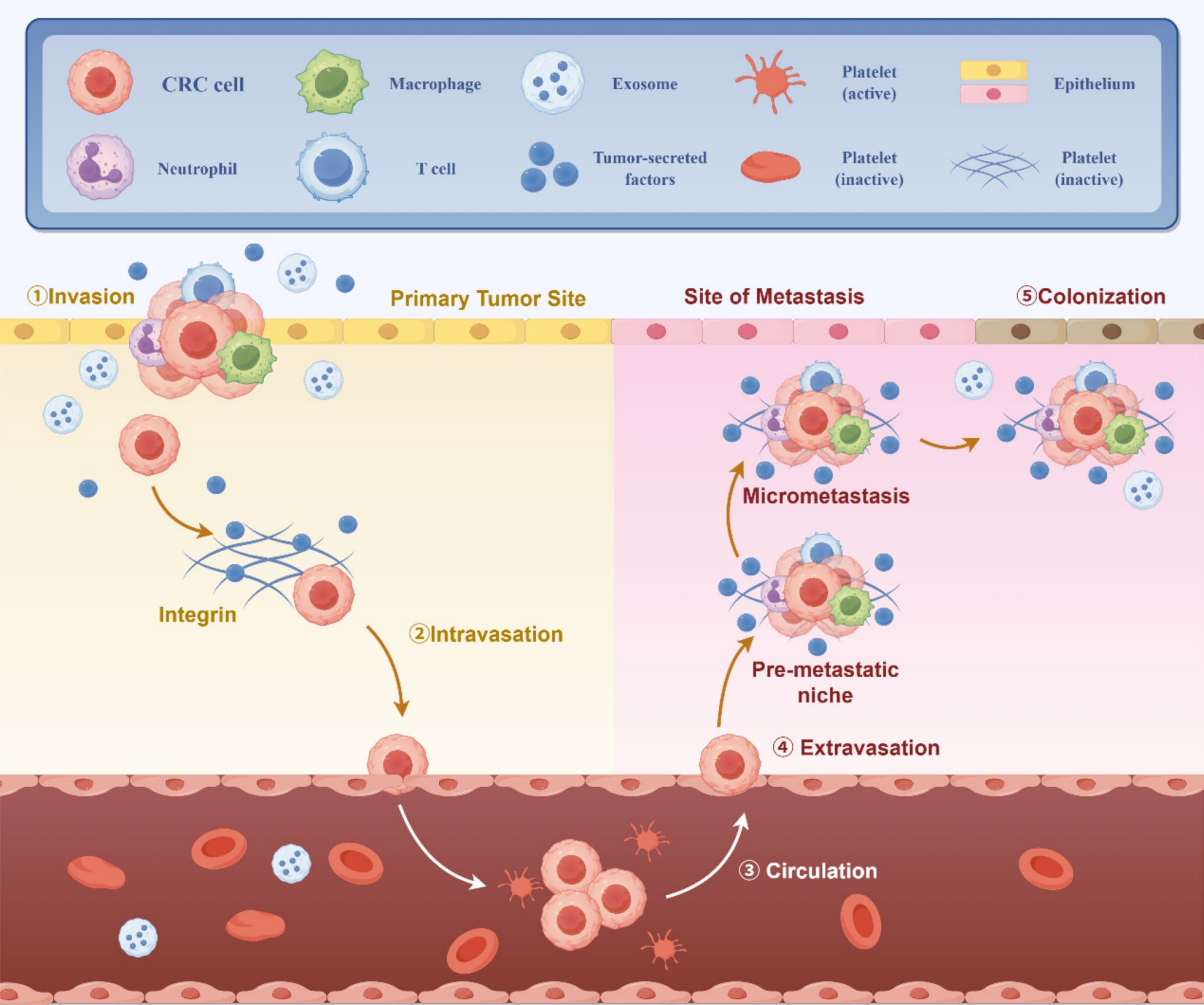
The illustration of CRC cells metastasizing through blood vessels. CRC cells cooperatively invade primary tumor sites in conjunction with macrophages, neutrophils, and T cells, where tumor-secreted factors subsequently bind to inactive platelets via integrins, thereby enabling CRC cell entry into the circulation and subsequent metastatic colonization of distant sites. Created in https://www.figdraw.com. ID: WRIIRbdd76

**Fig. 2:**
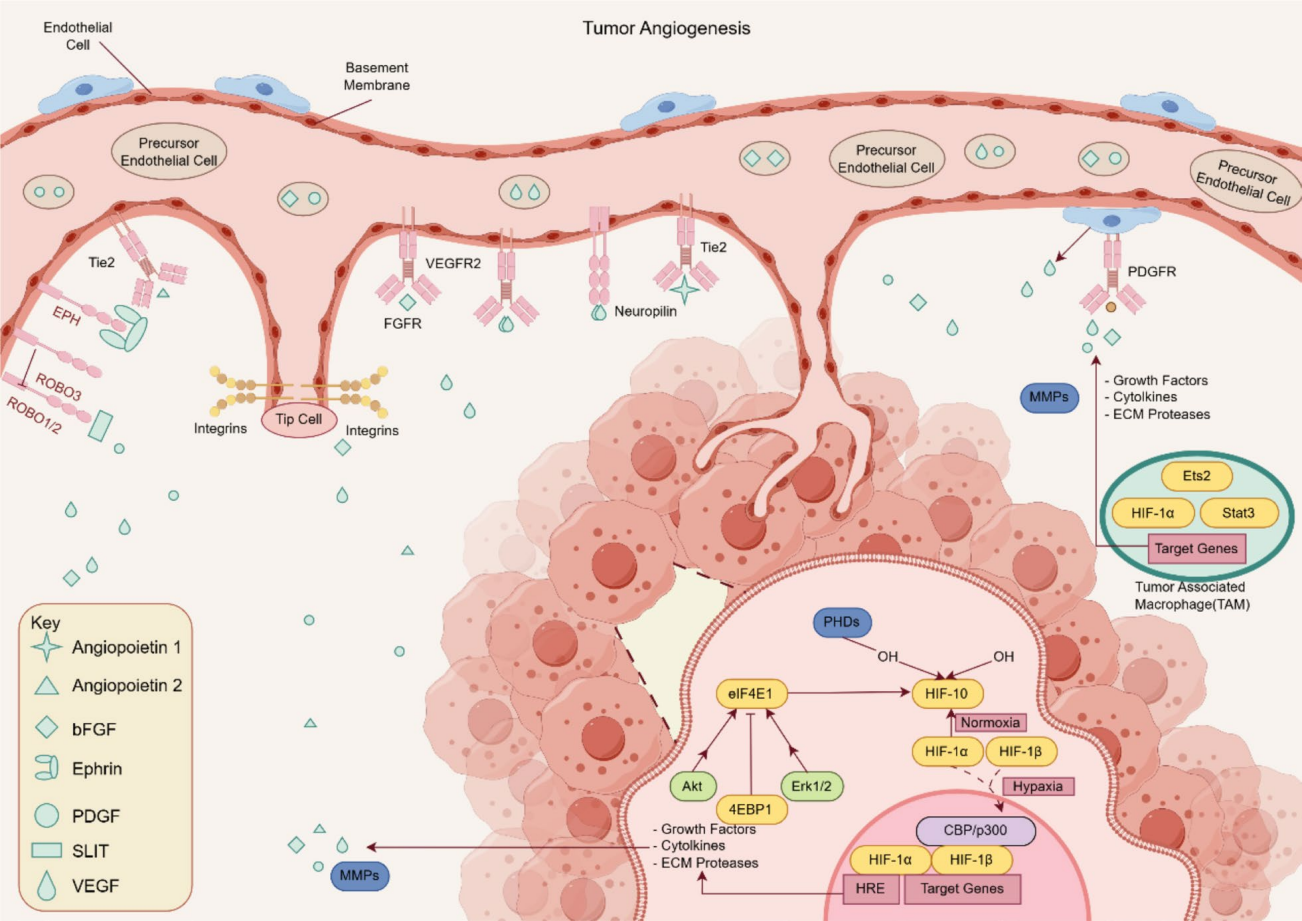
The process and principle schematic diagram of angiogenesis in the tumor microenvironment of CRC. The CRC cell mass modulates the expression of genes such as PHDs and elF4E1, thereby influencing Growth Factors, Cytokines, and ECM Proteases. This regulation subsequently controls the production of MMPs, which generate Angiopoietin 2, bFGF, PDGF, and VEGF. These factors act on blood vessels, inducing the formation of new vasculature within the tumor microenvironment. Created in https://www.figdraw.com. ID: WWOSS37778

In the process of tumor metastasis, for cancer cells, the phenomenon of autophagy and apoptosis inhibition will occur, so that the cells acquire more ability of division and proliferation. Autophagy indicates the process in which cytoplasmic proteins or organelles are encapsulated into vesicles of cells, and then form autophagolysosomes with lysosomes and degrade their contents. Moderate autophagy can effectively promote cellular growth, while excessive autophagy will kill cells. Apoptosis is the programmed death of cells. If cells show senescence or abnormality, the cell will start a self-destructive procedure leading to die naturally. The regulatory mechanism in the cancer cell has mutated so that apoptosis cannot occur. Tumor cells accumulate nutrients through angiogenesis, and then undergo epithelial-mesenchymal transition (EMT). Angiogenesis provides tumor cells with sufficient nutrients to accelerate the growth of tumor cells. The blood vessels surrounding the tumor are weaker than the walls of ordinary blood vessels, which allows tumor cells to pass through the wall into the blood and metastasize [10] (Fig. 1). Folkman found that solid tumor growth is always followed by neovascularization and suggested that tumor angiogenesis is necessary for tumor progression [11, 12]. Tumor vessels are not identical to normal vessels in morphology or function [12] High expression of pro-angiogenic factors by tumor cells acts on the vascular network to supply nutrients and oxygen [13] (Fig. 2). EMT refers to the process of converting cancer cells from epithelial cells to mesenchymal ones. This makes cancer cells lose epithelial characteristics such as intercellular adhesion and cell polarity, and acquire mesenchymal traits, including movement, invasion, and resistance to apoptosis. EMT changes the type of tumor cells, improves the ability to degrade the matrix, and weakens the intercellular binding force. Therefore, the transformed tumor cells have stronger ability to metastasize and invade, and are more likely to survive in the blood, which is related to drug resistance. EMT makes tumor cells detach from the primary or metastatic foci to form new secondary metastatic foci. Therefore, EMT is considered to be an important process for tumor cells to spread into blood circulation [14, 15]. Mitochondria also plays an important role in the process of tumor metastasis, involving in many important biological processes in the cell, it can provide energy for cell proliferation, regulate cell metabolism, control the generation of ROS [16] and influence the apoptosis of cancer cells through mitochondrial apoptosis [17]. The TME is a heterogeneous collection of cancer cells, cancer-associated fibroblasts (CAFs), immune/inflammatory cells [e.g., tumor-associated macrophages (TAMs), T cells, natural killer (NK) cells, myeloid-derived suppressor cells (MDSCs)], adipocytes, neurons, endothelial cells, secreted factors, and the extracellular matrix (ECM) that they secrete and mold into the extracellular space [18]. A series of cytokines, chemokines, growth factors, exosomes, and other signaling molecules interact with each other and constitute a network within the TME to give tumor the ability to sustain and survive the increased stress, leading to cancer metastasis, immune suppression, abnormal angiogenesis, and drug resistance [19–23]. CAFs are key components of the TME with diverse functions, including matrix deposition and remodeling, reciprocal signaling interactions with cancer cells, and crosstalk with infiltrating immune cells [24]. CAFs play a protumorigenic role in CRC by secreting factors to sustain cell proliferation, evade cell death, and recruit immune cells, making CAFs a potential target for. Therefore, to effectively suppress tumors metastasis, it is crucial to induce autophagy and promote apoptosis in tumor cells, thereby inhibiting their proliferative, migratory, and invasive capabilities. Furthermore, targeting tumor angiogenesis and suppressing tumor cell EMT are essential strategies. Tumor metastasis can also be suppressed by regulating the function of tumor cell mitochondria and TME [10].

**Fig. 3:**
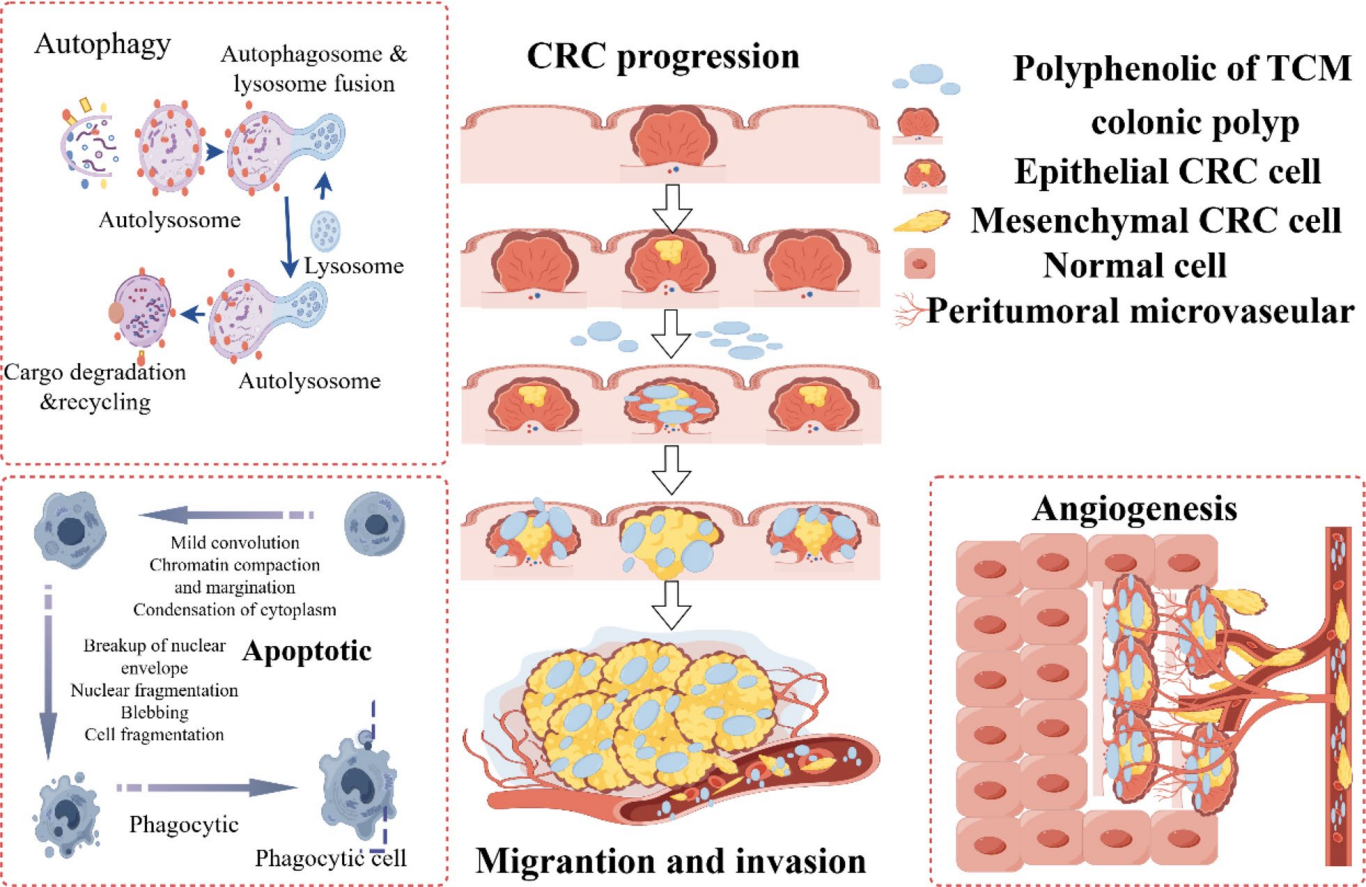
The schematic diagram of polyphenolic active compounds from Chinese herbs inhibiting colon cancer metastasis and invasion. The malignant progression of colonic polyps into colorectal cancer (CRC), followed by the migration and invasion of CRC cells into blood vessels, can be counteracted by polyphenolic compounds from TCM. These polyphenolic agents exert anti-angiogenic effects, promote autophagy and apoptosis in CRC cells, and suppress their migration and invasion. Created in https://www.figdraw.com. ID: WYATSdee87

With the continuous advancement in understanding the mechanisms of cancer, numerous therapeutic approaches for colon cancer have been established, including chemotherapy, radiotherapy, surgery, gene therapy, and immunotherapy. However, chemotherapy and radiotherapy are often associated with severe adverse effects. Although surgery can significantly prolong patient survival, it may substantially compromise postoperative quality of life. In clinical practice, gene therapy and immunotherapy have yet to be widely adopted. Traditional Chinese Medicine (TCM) can serve as an adjunct to chemotherapy and radiotherapy, alleviating severe adverse reactions, improving quality of life, and potentially enhancing therapeutic efficacy [25, 26]. Additionally, some clinically tested naturally anti-cancer bioactive products include vinblastine, vincristine, podophyllotoxin, paclitaxel (taxol) and camptothecin are employed as a complementary treatment modality [27]. Few secondary plant metabolites such as flavonoids, phenolics, terpenoids, saponins, quinones, and alkaloids have shown potent chemoprotective activity against CRC cells through triggering apoptosis and cell cycle arrest, modulation of tumour-suppressive microRNA, inhibition of oncogene and anti-apoptotic factor [28]. This review focuses on the latest advancements in the use of polyphenolic compounds from TCM for the treatment of colon cancer metastasis (Fig. 3).

**Fig. 4:**
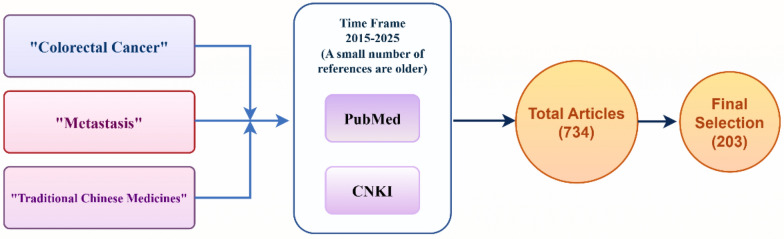
Flowchart of the article selection process. Articles were retrieved from PubMed and China National Knowledge Infrastructure (CNKI) using the keywords “Colorectal Cancer,” “Metastasis,” and “Traditional Chinese Medicines” published within the last ten years. A small number of references were older. A total of 734 articles were identified. After screening for relevance to the research topic, language (English and Chinese), and full-text availability, 203 articles were finally included. Created in https://www.figdraw.com. ID: OTOWO0e216

A total of 734 articles were initially identified from PubMed and China National Knowledge Infrastructure (CNKI) using keywords such as “traditional Chinese medicine,” “Colorectal Cancer,” and “Metastasis,” focusing primarily on studies published within the last ten years. A limited number of older references were also included. The articles were then screened according to their relevance to the research topic, language (English and Chinese), and full-text availability. After applying these criteria, a final selection of 203 articles was made (Fig. 4). Consequently, this review seeks to elucidate the role and mechanisms of TCM Polyphenolic in regulating metastasis of CRC. The objective is to enhance the clinical application of TCM Polyphenolic and provide a theoretical foundation for their use in metastasis of CRC.

## Mechanisms of polyphenolic active substances in TCM on the metastasis of CRC

**Fig. 5:**
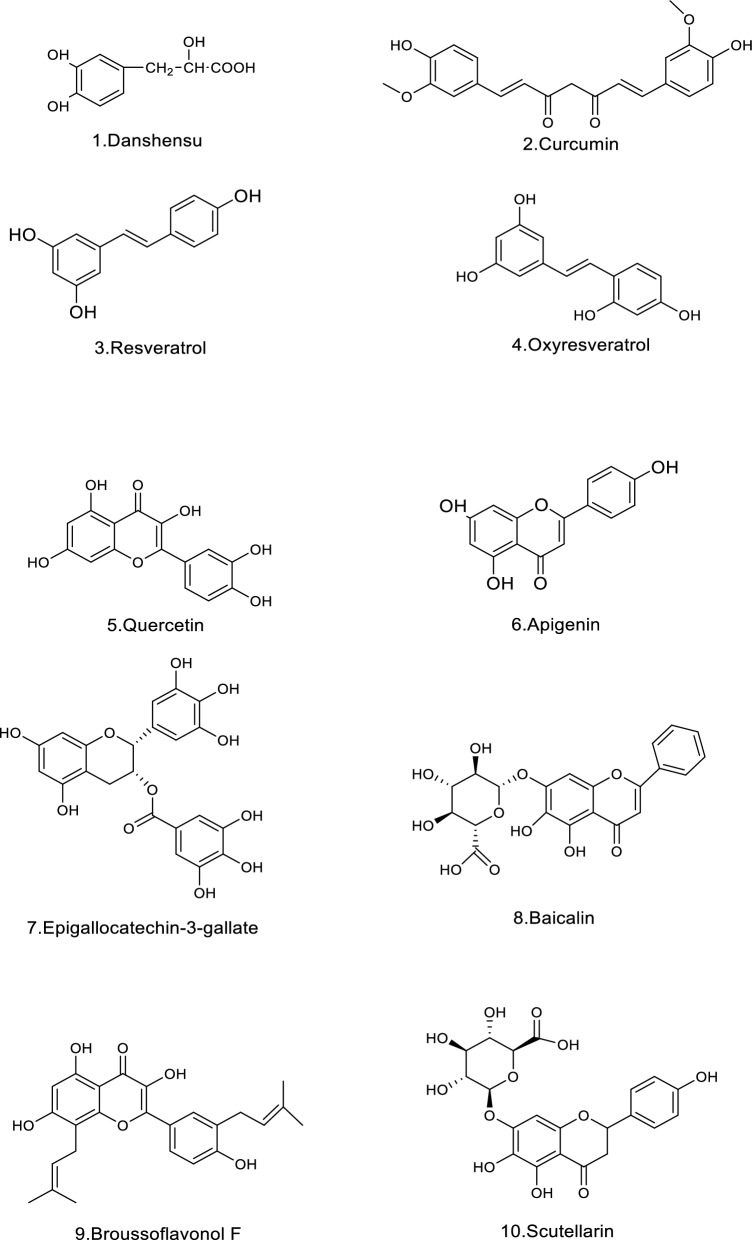

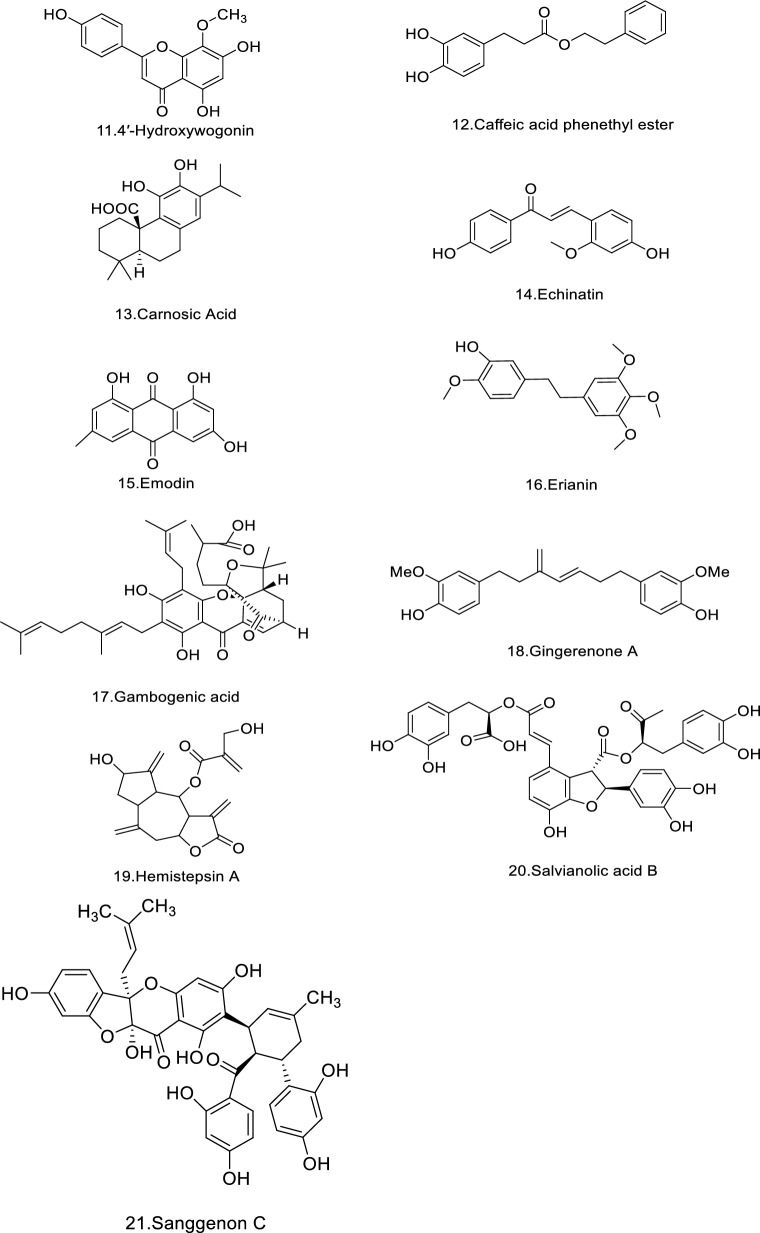
Chemical structures of polyphenols. Created in ChemDraw23

**Fig. 6:**
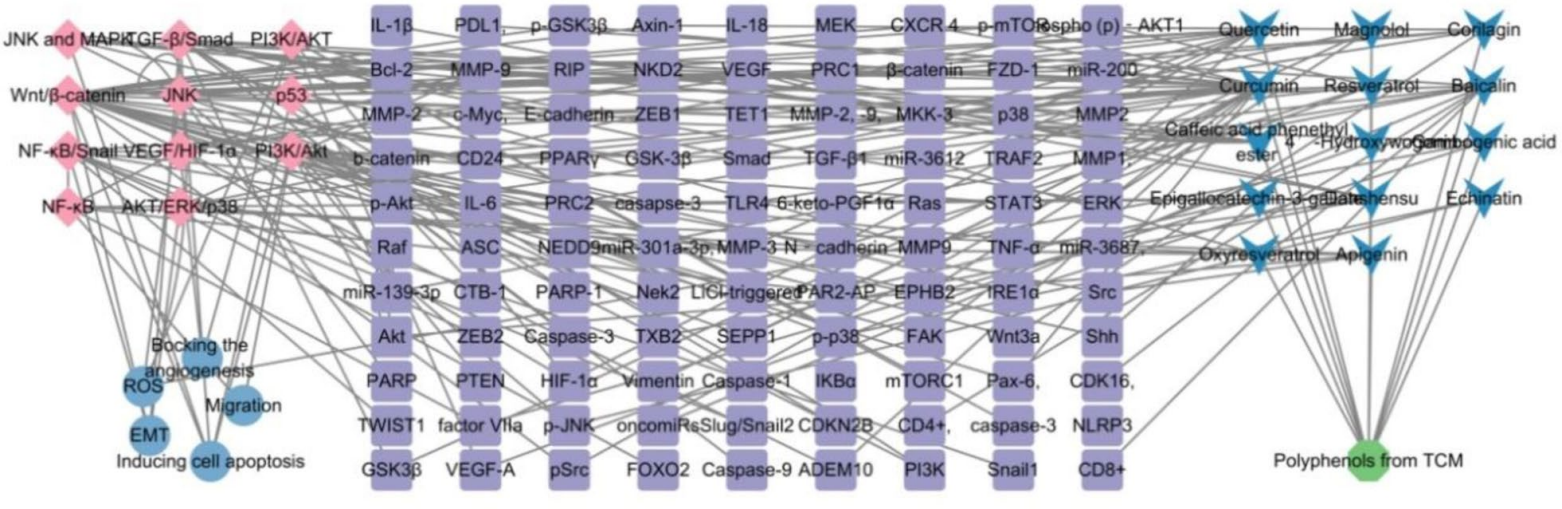
Network diagram illustrating the relationships among polyphenolic compounds, their targets, signaling pathways, and mechanisms of action. In this figure, V-shaped symbols represent polyphenolic compounds, square nodes indicate action targets, diamond-shaped nodes denote signaling pathways, and circular nodes represent mechanisms of action in inhibiting CRC. Created in Cytoscape

Colorectal cancer cell can migrate to other tissues and organs through lymphatic and blood circulation. Direct spread mainly include liver, lung and gastric and those with liver as the primary metastasis account for 30–60% and the five-year survival rate after metastasis is only 20% [29, 30]. According to the pathological process of CRC metastasis, it has been reported that CC metastasis is inhibited by TCM through different mechanisms. This review has summarized the reported effective mechanisms of TCM, such as inhibiting the EMT, inhibiting angiogenesis, promoting apoptosis, induce autophagy, inhibiting the proliferation migration, invasion and other functions and regulating the function of tumor cell mitochondria, shown in Table 1 and Fig. 5, 6.

**Table 1:** Polyphenolic compounds, their plant sources, primary mechanisms of action major molecular targets, and the experimental models

No.	Polyphenolic compound	Plant sources	Function	Mechanisms of action	Molecular targets	Experimental models	References
1	Danshensu	*Salvia miltiorrhiza*	Inhibit the EMT	TGF-β/Smad signaling pathwayt	IL-6, TNF-α, IL-1β, VEGF	SW620	[38]
			Anti-angiogenic	Alleviation of tumor cell hypoxia following treatment with danshensu + RT	TXB2, 6-keto-PGF1α,	Female C57BL/6J mice	[40]
2	Curcumin	*Curcuma longa*	Inhibit the EMT	NKD2-Wnt-CXCR4 signaling pathway	NKD2, CXCR 4	SW620	[44]
			Inhibit the EMT	FAK/p-Src axis	pSrc, FAK	Colon 26-M01 cell line	[45]
			Inhibit the EMT	Wnt signaling pathway	p-mTOR, p-Akt, β-catenin, p-GSK3β	Hep3B cells	[47, 48]
			Inhibit the EMT	TGF-β, Wnt, and PI3K/Akt/mTOR	ADEM10, calmodulin, EPHB2, HDAC4, CD24 and SEPP1	–	[52]
			Inhibit the EMT	PRC1/PRC2	miR-200	HCT116, SW480, male athymic nude mice	[53]
			Inhibit the EMT	Hedgehog signaling pathway	Vimentin, b-catenin	LN 229, U 251, female nude mice	[54]
			Inhibit the EMT	TET1-NKD2-WNT signal pathway	Pax-6, TET1, NKD2	rHCT-116	[55]
3	Resveratrol	*Rubus (raspberry)*	Inhibit the EMT	TGF-β1/ Smads signaling pathway	MMP2, MMP9, Snail/E-cadherin	LoVo	[41]
			Inhibit the EMT	AKT/GSK‐3β/Snail signaling pathway	N‐cadherin, phospho (p)‐AKT1, p‐GSK‐3β, Snail	SW480, SW620, BalB/c (nu/nu) mice	[67]
		*Rubus (raspberry)*	Inducing cell apoptosis	ABC-transporters proteins (MDR1, MRP1, and BCRP)	GST, CYP3A4, hPXR	Caco-2, CEM/ADR 5000	[110]
			Inducing cell apoptosis	AKT/STAT3 signaling pathway	AKT1, AKT2	DLD1, HCT15 HCEC	[111]
			Inducing cell apoptosis	PI3K/Akt signaling pathway	BMP7	HCT116	[99]
			Inducing cell apoptosis	NF-κB pathway	NLRP3, ASC, Caspase-1, IL-18, IL-1βTNF-α and IL-6	HT29	[94]
4	Oxyresveratrol	*Smilax china*	Inhibit the EMT	p53/miRNAs mechanism	miR-145, miR-200c	TW01, TW06, HONE-1, NOD/SCID mice	[63]
			Inhibit the EMT	Snail/E-cadherin, TGF-β-induced	miR-3687, miR-301a-3p, miR-3612	HT-29, HCT116	[66]
5	Quercetin	*Scutellaria baicalensis*	Inhibit the EMT	MKK-3/p38/NF-κB Pro-oncogenic signaling pathway	MMP-9, MKK-3	HCT116, SW480	[68]
			Inhibit the EMT	PLAU and its downstream pathways	MMP1, MMP3, MMP9	–	[69]
		*Ferula resin*	Bocking the angiogenesis	Cyclooxygenase-2	P300 Signaling,	MDA-MB-231, MCF7, DLD-1, HUVECs	[136]
		*Ferula resin*	Regulating the function of tumor cell mitochondria and controlling the generation of ROS	Suppressing ROS, PI3K/AKT pathway	HIF-1α	LOVO, HT-29, Female BALB/c nude mice	[170]
				Inhibition of COX-2	COX-2	HT29, HCT15, IEC-6	[173]
6	Apigenin	*chamomilla*	Inhibit the EMT	NF-κB/Snail pathway	NF-κB, Snail	Bel-7402	[60]
7	Epigallocatechin-3-gallate	*Crataegus spp*	Inducing cell apoptosis	Hedgehog/phosphoinositide 3-kinase pathways	Shh, PI3K	SW480, HCT-116, Caco2, Female BALB/c nude mice	[98]
			Inducing cell apoptosis	downregulates the cell cycle regulator Nek2	Nek2	HCT-116	[109]
			Inducing cell apoptosis	Wnt/β-Catenin Pathway	LiCl-triggered	DLD-1, SW480	[106]
			ROS	Nrf2-inhibited mitochondrial ROS accumulation	Nrf2	Caco2, AOM/DSS mice	[177]
8	Baicalin	*Scutellaria*	Inducing cell apoptosis	TLR4/NF-κB signaling pathway	PDL1, CD4^+^, CD8^+^	HCT-116, CT26, female Balb/c mice	[93]
			Inducing cell apoptosis	miR-139-3p/CDK16 axis	CDK16, miR-139-3p	SW480, HCT-116, CT 26, Male BALB/c nude mice	[112]
			Inducing cell apoptosis	Reduces c-Myc expression	c-Myc, oncomiRs	HT-29	[113]
			Inducing cell apoptosis	TGF-β/Smad pathway	TGF-β1	FHC, RKO, HCT116, BALB/c nude mice	[102]
9	Broussoflavonol F	*Macaranga genus*	Blocking the angiogenesis	HER2-RAS-MEK-ERK pathway	RAS, p-BRAF, p-MEK, p-Erk	HCT116, LoVo	[126]
10	Scutellarin	*Scutellaria altissima leaves*	Blocking the angiogenesis	Targets ephrinB2	ephrinb2	SW620, HCT116, LOVO, HT29, Balb/c nude mice	[127]
11	4′-Hydroxywogonin	*Scutellaria barbata*	Blocking the angiogenesis	PI3K/AKT pathway	VEGF-A	SW620, HIEC, HUVECs	[119]
12	Caffeic acid phenethyl ester	*honeybee propolis*	Blocking the angiogenesis	PI3K/AKT/mTOR Pathways	MMP-2, -9, VEGF	CT26, BALB/c male mice	[122]
13	Carnosic Acid	*Rosmarinus officinalis L*	ROS	Interference with STAT3 signaling pathway via generation of ROS	Mdm2, P53	HCT116, SW480, HT-29	[176]
14	Echinatin	*licorice (Glycyrrhiza species)*	ROS	JNK/p38 MAPK signaling pathway	Caspase + cells, p-JNK, p-p38	HCT116, HT29, HaCaT	[169]
15	Emodin	*Rheum palmatum, Polygonum cuspidatum* and* Polygonum multiflorum*	ferroptosis	NCOA4-mediated ferritinophagy and inactivation of the NF-κb pathway	Caspase-3	HT-29, RKO, HCT-15 SW620, female BALB/c Nude mice	[160]
16	Erianin	*Dendrobium nobile Lindl., Dendrobium JournalPre-proof chrysotoxum Lindl., Dendrobium fimbriatum Hook. var*	Ferroptosis	Inducing autophagy-dependent ferroptosis	KRAS^G13D^	HCT116, LoVo, Male BALB/c mice	[161]
17	Gambogenic acid	*Garcinia hanburyi* Hook.f	ROS	ROS/IRE1α/ JNK signaling pathway	p-IRE1α, IRE1α, GRP78	HCT116, SW620, DLD-1, CCD841	[165]
18	Gingerenone A	*Zingiber officinale Roscoe*	Ferroptosis	SLC7A11 signaling pathway	SLC7A11	NCM460, HCT-15, HCT-116, BALB/c nude mice	[152]
19	Hemistepsin A	*Hemistepta lyrata Bunge*	ROS	Inhibits the PDK1 activity	PDK1 caspase‐3, ‐9	SW480, RKO, Male BALB/c mice	[179]
20	Salvianolic acid B	*alvia miltiorrhiza*	ROS	Downregulates P-gp expression and promote tumor cell apoptosis	Bcl-2, Bax, P-gp	HCT-8, HCT-8/VCR	[182]
21	Sanggenon C	*Morus cathayana*	ROS	Inhibition of the iNOS and decreased NO production	caspase-9	LoVo, HT-29, SW480, male BALB/c-nu/nu nude mice	[171]

### Inhibiting EMT of colon cancer cells by polyphenolics TCM

**Fig. 7:**
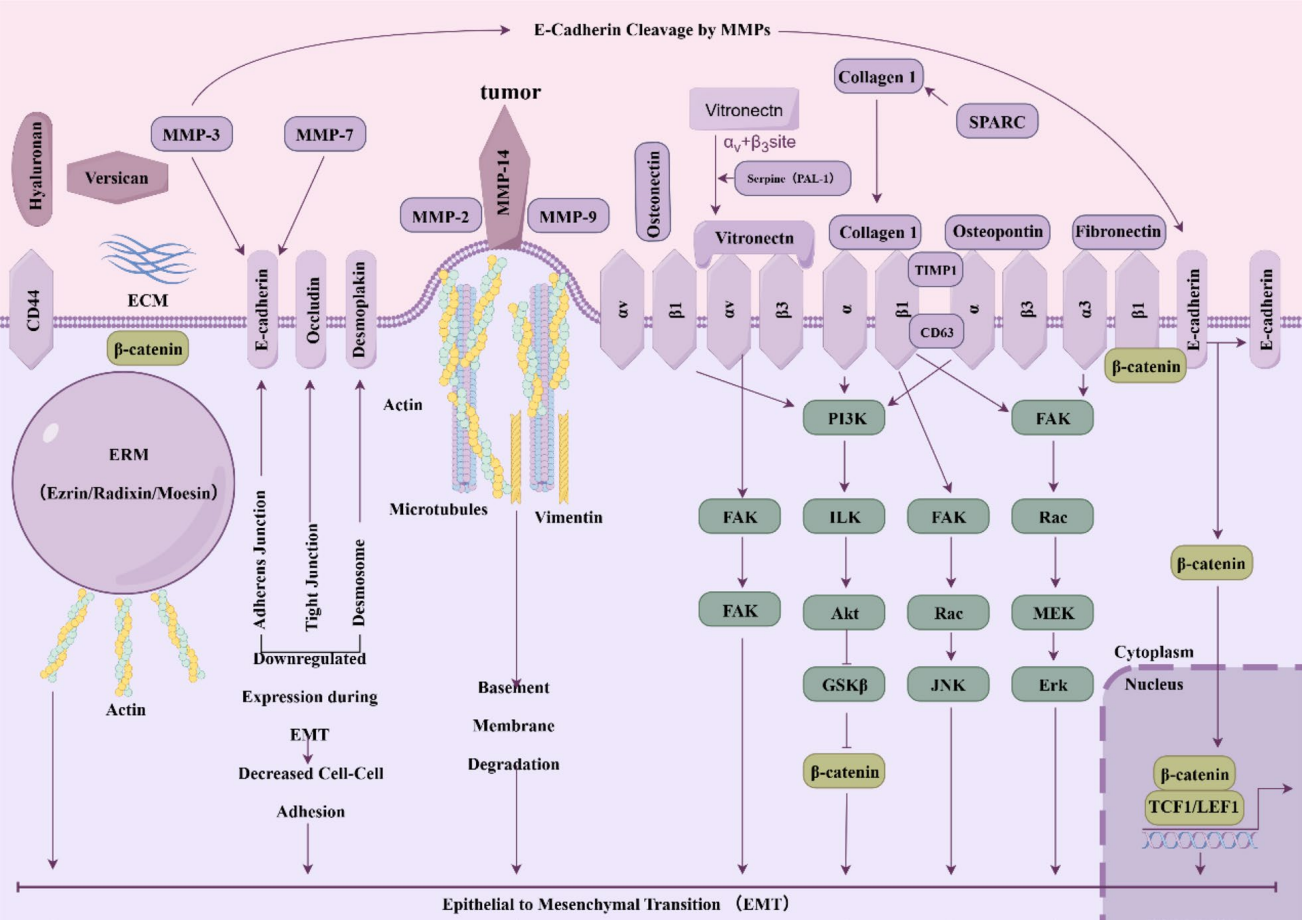
Contribution of extracellular matrix to EMT. The upregulation of MMPs expression in tumor cells acts on E-cadherin and β-catenin, leading to weakened adherens junctions, decreased cell–cell adhesion, and promoting the EMT process in CRC. Vitronectin facilitates EMT progression by acting on pathways such as FAK, JNK, PI3K/Akt/β-catenin, MEK, and Erk. Created in https://www.figdraw.com. ID: OIYSI99f9d

EMT is a critical biological process in the progression and metastasis of colorectal cancer. During EMT, epithelial cells lose their polarity and cell–cell adhesion properties, primarily through the downregulation of E-cadherin expression and the disruption of intercellular junctions. Concurrently, mesenchymal markers such as N-cadherin and Vimentin are upregulated, and the cytoskeleton is reorganized to form pseudopodia, enhancing cellular motility. Additionally, cancer cells secrete matrix metalloproteinases (MMPs), which degrade the extracellular matrix (ECM), facilitating cell migration and invasion [31]. The regulation of EMT involves multiple signaling pathways and transcription factors. Key transcription factors include: Snail family (e.g., Snail1, Snail2): These factors suppress E-cadherin expression. ZEB family (e.g., ZEB1, ZEB2): They inhibit the expression of epithelial genes while promoting the expression of mesenchymal genes. Twist family (e.g., Twist1, Twist2): These factors drive the mesenchymal phenotype. Major signaling pathways implicated in EMT include: TGF-β pathway: TGF-β is a primary inducer of EMT, activating the process through both Smad-dependent and Smad-independent mechanisms. Wnt/β-catenin pathway: Accumulation of β-catenin in the nucleus leads to its interaction with TCF/LEF transcription factors, promoting the expression of EMT-related genes. Notch pathway: Notch signaling promotes EMT through downstream effectors such as Hes1 and Hey1.PI3K/AKT pathway: This pathway stabilizes Snail and Twist transcription factors by inhibiting GSK-3β. MicroRNAs (miRNAs) also play a regulatory role in EMT: miR-200 family: These miRNAs negatively regulate EMT by suppressing ZEB1 and ZEB2 expression. miR-21: This miRNA promotes EMT-related signaling pathways, including TGF-β and PI3K/AKT [31–34] (Fig. 7). Recently, the possibility that EMT may encompass a range of intermediate states has been proposed. This phenotype, referred to as ‘partial EMT’ or ‘hybrid EMT’, describes cancer cells that exhibit both mesenchymal and epithelial characteristics due to internalization of the epithelial markers rather than transcriptional repression of the proteins [35, 36]. More than 90% of human CRC cell lines exhibit partial EMT, a status that favors the formation of cell clusters during CRC dissemination [37, 38]. Thus, EMT is a promising target to prevent primary tumors acquiring invasive properties or to prevent recurrence after resection of the tumors and metastases. Development of drugs that target the EMT directly is challenging due to the plasticity and heterogeneity of the various pathways involved. However, potential therapeutic strategies may be to: (i) combine EMT inhibitors with conventional chemotherapy agents to overcome pharmacological resistance in mCRC; and (ii) use EMT inhibitors in the adjuvant setting to reduce recurrence after resection of the tumors [17, 39].

#### Polyphenols targeting the TGF-β/Smad pathway in EMT

Danshensu, a polyphenolic compound derived from the TCM herb Salvia miltiorrhiza, was demonstrated by Cao et al. to attenuate CRC metastasis and chemoresistance via dual regulatory mechanisms. Specifically, Danshensu suppresses the activation of the TGF-β/Smad signaling pathway and inhibits platelet activation and secretory activity within the tumor microenvironment, particularly restraining the release of tumor-promoting factors such as IL-6, TNF-α, IL-1β, and VEGF. These cytokines are associated with driving EMT and fostering chemoresistance in cancer cells [38, 39]. In this study, although 25 μM of Danshensu could not directly inhibit the invasion of SW620 cacner cells, it was able to interfere with the activation of platelets by SW620 cancer cells and achieved the effects of reducing plateletinduced SW620 cancer cell migration and chemoresistance. The pretreatment of 25 μM Danshensu attenuated the release of IL-1β, IL-6, VEGF and TNF-α in the co-cultured platelet-tumor cell system, which reduced the effect of platelets on EMT and drug resistance of tumor cells. The underlying mechanism that Danshensu interfered with the interactions between platelets and tumor cells may be associated with the decreased activation of TGF-β/Smad signaling pathway. Therefore, Danshensu may emerge as an effective compound that potentially exerts anti-tumor activity through interfering with platelets. At the molecular level, Danshensu downregulates EMT-related proteins (e.g., N-cadherin and Vimentin) while significantly enhancing the expression of the epithelial marker E-cadherin, effectively reversing EMT progression [40], by blocking EMT dynamics, Danshensu disrupts tumor cell plasticity, thereby impeding metastatic dissemination and restoring chemotherapeutic sensitivity in colorectal cancer models. The alleviation of tumor cell hypoxia following treatment with danshensu alone was not observed in this study, the reason may be associated with drug dose or frequency of drug administration, so additional study is required to clarify this point.

Resveratrol, a non-flavonoid polyphenolic compound derived from traditional medicinal plants such as Rubus (raspberry) and other botanical sources, has been characterized as an EMT inhibitor. Ji et al. demonstrated that resveratrol significantly suppresses metastatic dissemination in CRC models, as evidenced by its inhibition of both tail vein-injected CRC cell colonization and metastasis from orthotopic colon tumors in mice. Mechanistic investigation revealed resveratrol’s ability to abrogate TGF-β1-induced EMT through suppression of the canonical Smads signaling pathway [41].

Magnolol, a principal polyphenolic bioactive component isolated from Magnolia officinalis bark [42], was demonstrated by Chei et al. to suppress EMT progression in CRC via TGF-β signaling pathway interception and cell cycle redistribution [43].

#### Polyphenols targeting the Wnt/β-catenin pathway in EMT

Curcumin, a polyphenolic compound derived from the rhizomes of Curcuma longa, has demonstrated multifaceted mechanisms to inhibit the EMT in CRC and regulate metastasis. Studies by Zhang and Li revealed that curcumin upregulates the expression of the epithelial marker E-cadherin while downregulating mesenchymal markers including vimentin, N-cadherin, and β-catenin [44, 45]. In the current study, different concentrations of curcumin were used for 24 h in SW620 cells. Founding that curcumin has a strong inhibitory effect on the Wnt signaling pathway and EMT. However, these anti-metastatic effects remain to be elucidated. Katoh et al. further demonstrated that curcumin enhances NKD2 expression [46], thereby suppressing Wnt signaling pathway activation [47, 48] and CXCR4 expression [49, 50], ultimately inhibiting tumor cell EMT and reducing hepatic, pulmonary, and peritoneal metastases [51]. In the present study, migration triggered by TPA was found to be suppressed by curcumin, confirming that curcumin inhibits TPA-induced Wnt signaling activation. In addition, curcumin was observed to suppress tumor growth and microvessel density by blocking the Wnt signaling pathway in vivo. Mechanistically, curcumin disrupts cytoskeletal remodeling by suppressing the cofilin pathway [45] and attenuates focal adhesion signaling through inhibition of the FAK/p-Src axis [45]. Additionally, Bahrami et al. identified curcumin’s inhibitory effects on multiple oncogenic pathways including TGF-β, Wnt, and PI3K/Akt/mTOR [52], while Toden et al. revealed its capacity to downregulate polycomb repressive complexes (PRC1/PRC2) by reducing expression of BMI1, SUZ12, and EZH2 [53]. Meng et al. elucidated curcumin-mediated suppression of Hedgehog signaling to impede EMT progression [54], whereas Lu et al. described its ability to reverse EMT through epigenetic modulation of the TET1-NKD2-WNT signaling axis [55]. Although the finding was interesting, deep investigation on the transcriptional mediating effect of curcumin on TET1 and the demethylation function of TET1 on NKD2 should be performed in the future wok to better understand the relationship between the anti-resistant effect of curcumin and TET1-NKD2-WNT signal pathway. In addition, the mechanism should be verified in the in-vivo animal model and in the future work, the verification should be claimed. These collective findings underscore curcumin’s pleiotropic antitumor activities involving transcriptional regulation, epigenetic modification, and multi-pathway crosstalk in CRC metastasis control.

Corilagin, a distinctive tannin constituent abundant in Phyllanthus species [56], inhibits CRC metastasis through Wnt/β-catenin signaling pathway modulation and EMT driver signal blockade, as elucidated by Sun Tae Hwang et al. [57].

Gallocatechin, a polyphenolic compound extracted from Spatholobi caulis using ethyl acetate [58], was shown by Sun et al. to inhibit EMT-driven metastasis in CRC through β-catenin activation blockade [59].

#### Polyphenols targeting the NF-κB/Snail transcriptional axis in EMT

Apigenin, a naturally occurring flavonoid (polyphenolic) compound widely distributed in Scutellaria baicalensis, Perilla frutescens, and Matricaria chamomilla, among other species, has been shown by Tong and Qin et al., to suppress EMT progression, migration, and invasion in CRC through modulation of the NF-κB/Snail signaling axis. Mechanistically, apigenin inhibits NF-κB transcriptional activity, thereby reducing the expression of the downstream transcription factor Snail. This disruption leads to the upregulation of the epithelial marker E-cadherin and downregulation of the mesenchymal marker Vimentin, collectively attenuating EMT-driven tumor cell plasticity and metastatic behavior in CRC models [60, 61]. These findings highlight apigenin’s therapeutic potential in targeting signaling cascades critical to tumor progression and invasiveness.

The natural polyphenol genistein, derived from traditional Chinese medicinal plants including Flos Pueraria lobata and Astragalus membranaceus, was reported by Zhou et al. to reverse EMT via dual regulation of cadherin expression—promoting E-cadherin while suppressing N-cadherin—combined with modulation of EMT transcription factors (Snail1, Slug/Snail2, ZEB1, ZEB2, FOXO2, and TWIST1). Additionally, genistein enhances pro-apoptotic Bax/Bax-2 and caspase activities through Notch-1 pathway inhibition, subsequently suppressing both phosphorylated and total NF-κB expression to further negatively regulate EMT [62].

Oxyresveratrol, a structurally related compound isolated from the traditional herb Smilax china, exhibits comparable anti-EMT activity. Shen and Kim et al. reported its dual regulatory effects on the Snail/E-cadherin transcriptional axis and p53 activation via phosphorylation [63, 64], while concurrently inhibiting NF-κB signaling. Yan et al. elucidated oxyresveratrol’s epigenetic modulation of EMT, involving downregulation of oncogenic miRNAs (e.g., miR-3687, miR-301a-3p) and upregulation of tumor-suppressive miR-3612, thereby disrupting EMT-associated transcriptional networks [65, 66]. Although the mechanism that correlates EMT and some miRNAs, such as miR-3687 and miR-3612, needs further investigation, these results demonstrate that Oxyresveratrol may have the potential to inhibit metastasis in CRC.

#### Polyphenols targeting other kinase signaling pathways in EMT(AKT/ERK/p38)

Li et al. further identified that resveratrol attenuates EMT progression by targeting the AKT/GSK3β/Snail signaling axis, effectively restoring epithelial phenotypes [67]. It was confirmed that resveratrol suppressed the migration and invasion of colon cancer cells both in vitro and in vivo.

Quercetin, a flavonoid (polyphenolic) compound derived from Scutellaria baicalensis and other botanical sources, suppresses colorectal cancer metastasis through downregulation of MMP9 expression via the MKK-3/p38/NF-κB signaling axis, as evidenced by studies [68, 69]. Mechanistically, this phytochemical exerts anti-metastatic effects by dual modulation of extracellular matrix remodeling and EMT processes: it attenuates MMP-2 and MMP-9 proteolytic activities while normalizing the expression of EMT-related biomarkers. Specifically, quercetin upregulates epithelial marker E-cadherin and concurrently downregulates mesenchymal markers N-cadherin, β-catenin, and transcription factor snail, thereby inhibiting in vitro migratory and invasive capacities of CRC cells [70].

#### Polyphenols with complex or pleiotropic regulatory mechanisms

Notably, tannic acid from Galla Chinensis exhibits paradoxical EMT modulation: Barboura et al. found it reduces mesenchymal biomarkers (β-catenin and E-cadherin) while increasing epithelial markers (Slug, Snail, ZEB1, and N-cadherin), ultimately inhibiting CRC metastasis through this complex regulatory mechanism [71]. We have now discussed two plausible explanations: i) cell line-specific responses, where SW480 (primary tumor) and CT26 (murine) appear more sensitive to tannic acid, whereas SW620 (metastatic lesion) exhibits lower sensitivity and a more pronounced mesenchymal phenotype; and ii) the dynamic nature of EMT, where the timing of treatment and measurement may influence marker expression, potentially leading to divergent results depending on the phase of EMT examined.

Isovitexin (apigenin 6-C-glucoside), a natural flavonoid compound found in various medicinal plants including Cucurbitaceae fruits, tribulus terrestris, and bamboo leaves [72], has demonstrated anti-metastatic effects in CRC through EMT modulation. Zhu et al. revealed that isovitexin suppresses EMT and negatively regulates CRC metastasis by upregulating epithelial markers E-cadherin and ZO-1 expression [73].

### Inducing cell apoptosis

**Fig. 8:**
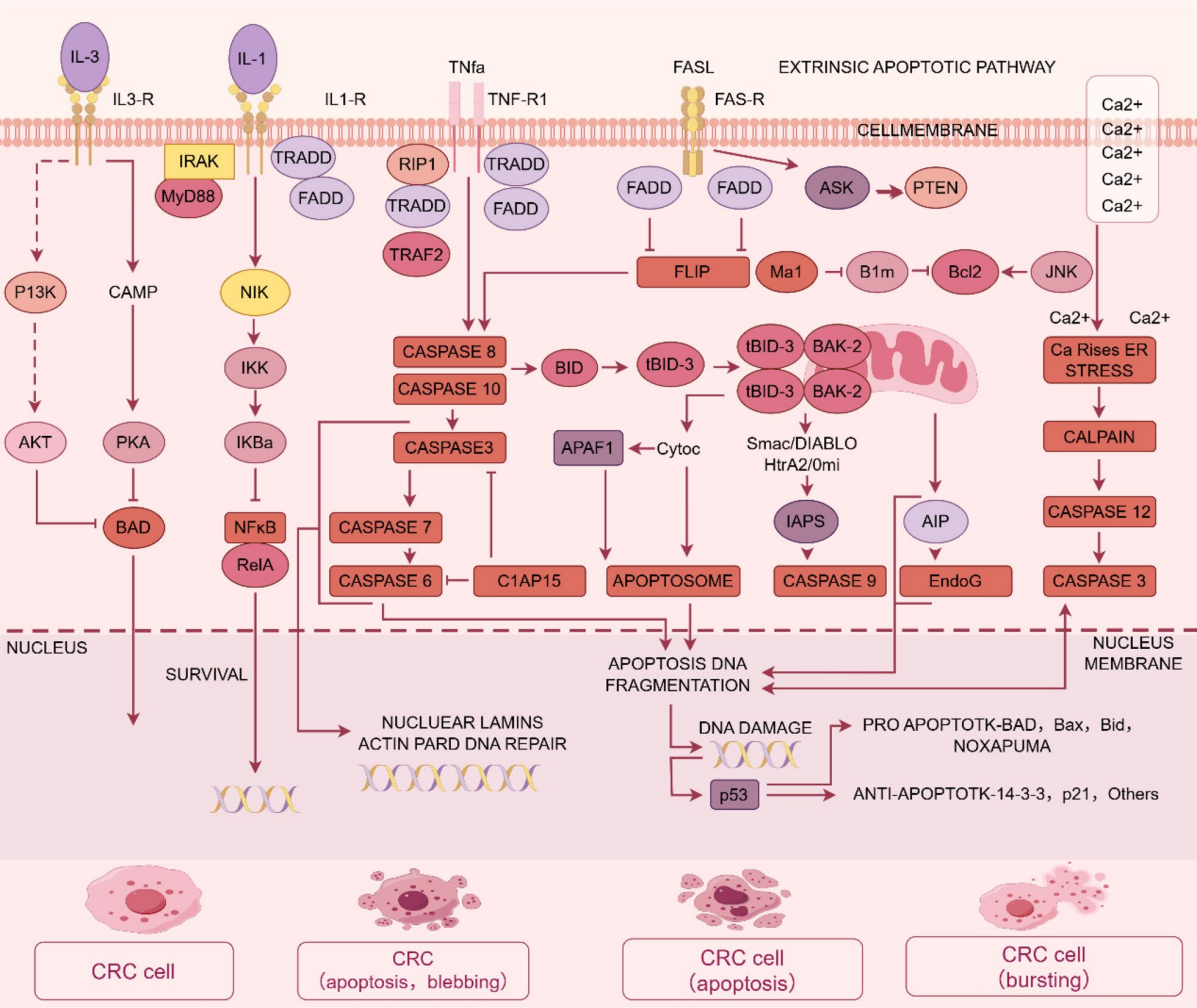
The extrinsic influences on cancer cell apoptosis. IL-3 regulates the PI3K/AKT pathway, IL-5 modulates the NF-κB pathway, and TNFα regulates the expression of the CASPASE family, acting on the p53 pathway to promote the apoptosis of CRC cells. Created in https://www.figdraw.com. DI: OIOAIf95a6

The regulation of apoptosis in CRC cells involves interdependent aberrations in key genes, signaling pathways, and post-transcriptional regulators. Central to CRC pathogenesis is the inactivation of tumor suppressor genes such as TP53 (whose mutations impair pro-apoptotic functions, facilitating tumor progression) [74]and APC (whose truncating mutations drive constitutive activation of the Wnt/β-catenin pathway, thereby suppressing apoptosis) [75]. Dysregulated signaling cascades further reinforce apoptosis resistance: Wnt/β-catenin signaling, hyperactivated by APC mutations, enhances survival and downregulates apoptotic effectors [76]; PI3K/AKT/mTOR pathway activation, often due to PI3K mutations or AKT overexpression, suppresses pro-apoptotic molecules (e.g., Bad, Bax) via phosphorylation-mediated inactivation [77]; MAPK/ERK signaling, triggered by oncogenic KRAS mutations, promotes proliferation while antagonizing apoptosis [78]. Apoptotic evasion is also mediated by imbalances in Bcl-2 family proteins, where elevated anti-apoptotic Bcl-2/Bcl-xL suppresses pro-apoptotic Bax/Bak, impairing mitochondrial cytochrome c release [79]. Additionally, extrinsic apoptosis pathways are compromised through Fas/FasL decoy receptor upregulation or TRAIL receptor silencing, enabling immune evasion [80]. Epigenetically, microRNAs critically modulate apoptotic sensitivity: miR-34a, transcriptionally regulated by p53, targets BCL2 and SIRT1 to restore apoptosis [81], whereas miR-21 sustains survival by repressing PTEN, activating PI3K/AKT signaling [82]. Collectively, these molecular perturbations establish a robust anti-apoptotic network that underlies CRC aggressiveness (Fig. 8).

#### Polyphenols targeting the JNK and MAPK pathway in inducing cell apoptosis

In addition to suppressing CRC metastasis through inhibition of EMT processes, curcumin demonstrates anti-metastatic effects via induction of cancer cell apoptosis [83–85]. Khaket et al. revealed that curcumin induces apoptosis in CRC cells through JNK activation-mediated pathways [86].

Caffeic acid phenethyl ester (CAPE), a major bioactive constituent of propolis extract, has been documented to demonstrate antimicrobial, antioxidant, anti-inflammatory, and cytotoxic properties [87]. Yahya et al. elucidated that CAPE treatment in HT-29 cells induced apoptosis through inactivation of critical molecules in the MEK signaling pathway, specifically p38 MAPK, ERK, and JNK. This mechanism resulted in cell cycle arrest at the G2/M phase and suppression of metastatic regulation in CRC cells [88].

Kee et al. revealed that quercetin activates intrinsic apoptotic pathways in CT-26 cells via regulation of ERK, JNK, and p38 MAPK signaling. Furthermore, it suppresses in vitro migration and invasion capabilities through MMP-2/9 inhibition and modulation of EMT-related biomarkers (E-cadherin, N-cadherin, β-catenin, and Snail) [89].

#### Polyphenols targeting the NF-κB pathway in inducing cell apoptosis

Baicalin, a bioactive flavonoid compound extracted from the dry roots of *Scutellaria baicalensis* Georgi, demonstrates diverse pharmacological properties including anticarcinogenic, antioxidant, antibacterial, and antitumor activities, with well-documented safety profiles in both animal models and human applications [90–92]. Song et al. demonstrated that baicalin suppresses CRC cell migration and invasion by inhibiting TLR4/NF-κB signaling pathway activation [93].

Additionally, Zhao et al. demonstrated in HT-29 cells that resveratrol attenuates LPS/ATP-induced pyroptosis—a pro-inflammatory form of programmed cell death—by downregulating pyroptosis-associated markers (NLRP3, ASC, Caspase-1, IL-18, IL-1β) and inflammatory cytokines (TNF-α, IL-6). This suppression was mediated through inhibition of the NF-κB pathway, further underscoring resveratrol's role in curbing inflammation-driven CRC metastasis [94].

Licochalcone A, a novel flavonoid isolated from Glycyrrhiza uralensis Fisch (licorice root), demonstrates antitumor efficacy by suppressing PD-L1 expression through NF-κB and Ras/Raf/MEK pathway blockade, thereby inhibiting CRC proliferation and promoting tumor cell death [95, 96].

#### Polyphenols targeting the PI3K/Akt pathway in inducing cell apoptosis

Epigallocatechin-3-gallate (EGCG), a natural polyphenolic compound derived from traditional Chinese medicinal herbs including Crataegus spp. and Eucommia ulmoides, exhibits regulatory effects on cancer metastatic activity through proliferation inhibition and induction of cell death [97]. Ding et al. demonstrated that EGCG modulates both the Sonic Hedgehog (Shh) pathway and PI3K/Akt signaling pathway by downregulating the protein expression levels of PI3K, phosphorylated Akt (p-Akt), Smoothened (Smo), and glioma-associated oncogene homolog 1 (Gli-1) [98].

Further mechanistic insights were provided by Zeng et al., who showed in HCT116 cells that resveratrol inactivates the PI3K/Akt signaling pathway via upregulation of BMP7 and partial suppression of PTEN phosphorylation [99].

#### Polyphenols targeting the TGF-β/Smad pathway in inducing cell apoptosis

Ellagic acid (EA), a polyphenolic compound derived from various botanicals including Paeonia lactiflora, Rubus idaeus, Terminalia chebula, Juglans regia, Commiphora myrrha, Eriobotrya japonica, Punica granatum, and Phyllanthus emblica [100], was shown by Zhao et al. to induce G0/G1 phase cell cycle arrest in HCT-116 cells. This effect was mediated through modulation of the TGF-β1/Smad3 signaling pathway, resulting in upregulation of cyclin-dependent kinase inhibitor CDKN2B and subsequent suppression of CRC metastasis [101].

Yang et al. provided comprehensive mechanistic evidence showing that baicalin: induces caspase-dependent apoptosis through elevated levels of cleaved Caspase-8, -9, -3, and PARP-1; triggers cell cycle arrest via suppression of cyclin D1, E1, B1, and p-Akt (Ser473); and attenuates transforming growth factor-β1 (TGF-β1)-induced cell migration in CRC models [102].

#### Polyphenols targeting the p53 pathway in inducing cell apoptosis

Proanthocyanidins, naturally occurring polyphenolic bioflavonoids, include cinnamtannin B-1 (CTB-1), a trimeric proanthocyanidin found selectively in traditional medicinal plants such as Cinnamomum spp [103]. Carrière et al. demonstrated that CTB-1 treatment in HCT-116 and Colo 201 cells enhanced WT p53 expression and phosphorylation at Ser6/Ser9 residues, subsequently inducing apoptosis while inhibiting CRC cell metastasis and invasion [104].

Tanshinone I, a key diterpenoid constituent of Salvia miltiorrhiza Bunge (Danshen), was identified by Lu et al. as exerting anti-metastatic effects in CRC through inhibition of the Aurora A-p53 axis [105].

#### Polyphenols targeting the Wnt/β-catenin pathway in inducing cell apoptosis

Chen et al. identified that EGCG suppresses colorectal cancer (CRC) metastasis through inhibition of the Wnt/β-catenin signaling pathway while promoting apoptosis in CRC cells [106]. This study was conducted exclusively in DLD-1 and SW480 cell lines, and the underlying mechanisms warrant further investigation using in vivo models.

#### Polyphenols targeting Other Signaling Pathways in inducing cell apoptosis

Furthermore, Ge et al. demonstrated that curcumin targets MACC1 to inhibit M2 polarization of tumor-associated macrophages (TAMs), thereby promoting cancer cell death and modulating CRC metastasis [107]. Notably, Liu et al. elucidated a comprehensive molecular mechanism wherein curcumin activates a ROS/KEAP1/NRF2/miR-34a/b/c signaling cascade to suppress colorectal cancer metastasis [108].

Wubetu et al. revealed that EGCG significantly downregulates the cell cycle regulator Nek2 (Never in mitosis gene A-related kinase 2), inducing G1/S phase arrest in HT-116 cells with a marked increase in the proportion of cells undergoing G1/S phase transition [109].

Mahmoud et al. demonstrated that treating Caco-2 cells with resveratrol for 48 h significantly downregulated the expression of multidrug resistance (MDR)-associated genes, including P-gp, MRP1, BCRP, cytochrome P3A4, glutathione transferase (GST), and hPXR. These findings elucidate resveratrol's dual mechanism of inhibiting both the efflux function and expression of ABC transporters (MDR1, MRP1, and BCRP), suppressing metabolic enzymes (GST and CYP3A4) and their regulatory gene (hPXR), and activating caspase-dependent apoptosis in MDR cells [110], positioning it as a potent phytochemical for modulating CRC metastasis. Complementary in vitro studies by Li et al. revealed that resveratrol directly binds to AKT1 and AKT2 in CRC cell lysates, and silencing AKT1/2 recapitulated resveratrol's effects by inhibiting cell proliferation, enhancing apoptosis, and inducing G1-phase cell cycle arrest [111]. Although resveratrol has a clear inhibitory effect on colon cancer cell proliferation and growth in vitro and ex vivo, these effects have not been confirmed yet in animal models and humans. Further research and clinical trials are warranted to fully elucidate the effects of resveratrol on human cancer.

Cai et al. further elucidated through in vitro studies that baicalin modulates CRC metastasis by upregulating miR-139-3p expression, consequently downregulating CDK16 expression [112]. The identification of the miR-139-3P-CDK16 axis mediated by baicalin as a potential pathway that regulates the viability and cell cycle in vitro suggested its vital role in mediating the oncogenesis of breast cancer. However, further experiments are required to fully clarify the mechanism by which baicalin represses the progression of colon cancer in vivo by regulating the miR-139-3p/CDK16 axis. Tao et al. identified that baicalin reduces c-Myc expression in CRC cells, leading to subsequent downregulation of oncomiRs (miR-63 to miR-70) and subsequent induction of apoptosis-mediated metastasis regulation [113].

### Blocking the angiogenesis

**Fig. 9:**
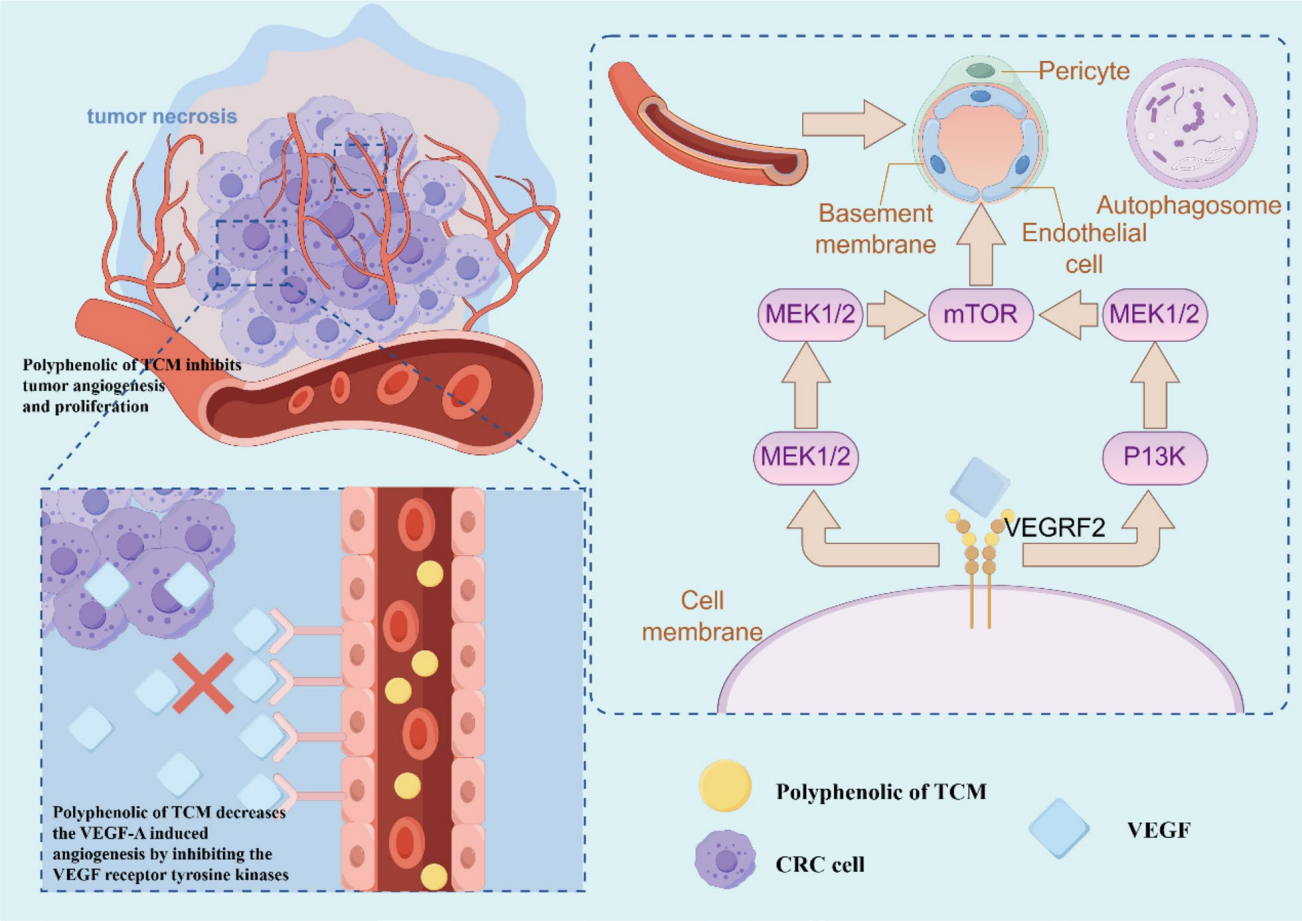
The mechanism diagram of polyphenolic compounds from TCM in inhibiting tumor angiogenesis. Polyphenolic compounds of TCM decrease the VEGF-A-induced angiogenesis by inhibiting the VEGF receptor tyrosine kinases, and VEGF2 exerts anti-angiogenic effects through PI3K, MEK1/2 and other pathways. Created in https://www.figdraw.com. DI: IITYW4000a

Angiogenesis provides tumor cells with sufficient nutrients to accelerate the growth of tumor cells. The blood vessels surrounding the tumor are weaker than the walls of ordinary blood vessels, which allows tumor cells to pass through the wall into the blood and metastasize [10]. Similar to other observed solid tumors, CRC development, pro gression, and metastasis depend heavily on angiogenesis [114, 115]. Numerous molecules participate in the process, such as growth factors (VEGF and epidermal growth factors [EGFs]), fibroblast growth factor (FGF)-2, transforming growth factor (TGF)-α and TGF-β, angiopoietins (Angs), platelet-derived growth factor (PDGF), membrane-bound factors (integrins, ephrins, cadherins, matrix metalloproteinases [MMPs], and hypoxia-inducible factor1 [HIF-1]) [116, 117] (Fig. 9).

#### Polyphenols targeting PI3K/AKT/mTOR pathways in bocking the angiogenesis

4′-Hydroxywogonin, a major natural polyphenol from Scutellaria barbata [118], reduces CRC cell viability and angiogenesis through downregulation of VEGF-A expression via disruption of the PI3K/AKT pathway [119].

Nobiletin, a natural polyphenol from Citrus reticulata, prevents CRC metastasis and progression by inhibiting mTORC1-mediated Akt signaling and angiogenesis [120].

3,3',4'-Trimethylellagic acid, a tannin from Sanguisorba officinalis L., modulates metastasis through Bax upregulation, caspase-3 phosphorylation, Bcl-2 downregulation, and inhibition of the VEGF/PI3K/AKT/mTOR pathway [121].

#### Polyphenols targeting VEGF/HIF-1α pathways in bocking the angiogenesis

Caffeic acid phenethyl ester (CAPE) from honeybee propolis decreases matrix metalloproteinase (MMP)-2 and -9 expression, inhibits VEGF production in CT26 cells, and reduces plasma VEGF levels in murine models, effectively suppressing colon cancer metastasis [122].

Orientin, a flavonoid found in Ocimum sanctum, exhibits anti-angiogenic activity by modulating HIF-1α and VEGFA [123].

Calebin A (CA), a polyphenol from Curcuma longa and Curcuma caesia rhizomes [124], suppresses angiogenesis and CRC metastasis by targeting HIF-1α and NF-κB [125].

#### Polyphenols targeting other signaling pathways in bocking the angiogenesis

Broussoflavonol F, a prenylated flavonoid isolated from the Macaranga genus, inhibits CRC metastasis by suppressing the HER2-RAS-MEK-ERK pathway and blocking angiogenesis [126].

Scutellarin, a natural polyphenolic compound derived from Scutellaria altissima leaves, suppresses human CRC cell growth, metastasis, and angiogenesis by targeting ephrinB2 [127].

Curcumin (a turmeric-derived natural polyphenol) combined with ( −)-epigallocatechin-3-gallate (EGCG, the most abundant active component in green tea) [128, 129] enhances bioavailability and exerts anti-angiogenic effects by blocking the JAK/STAT3/IL-8 signaling pathway, thereby inhibiting NEC migration to TECs [130].

#### Polyphenols with other mechanisms in bocking the angiogenesis

6-Gingerol, the primary bioactive compound in ginger, demonstrates anti-proliferative, anti-angiogenic, and anti-inflammatory effects in benzo[a]pyrene- and dextran sulfate sodium-induced CRC [131].

Galbanic acid (GBA), a sesquiterpene coumarin from Ferula resin, effectively inhibits tumor growth and angiogenesis in vivo [132]. Ellagic acid (EA), an ellagitannin-derived polyphenol, regulates CRC angiogenesis by modulating differentially expressed genes [133, 134].

Quercetin reduces MMP-2/9 expression and activity in primary (Caco-2) and metastatic (LoVo/LoVo/ADR) colon cancer cells [135]. However, further in vivo experiments and clinical trials are recommended. And significantly suppresses COX-2-mediated angiogenesis in endothelial cells treated with conditioned media from DLD-1 CRC cells [136].

### Suppressing proliferation, migration and invasion of CRC cells

#### Polyphenols targeting NF-κB Pathways in suppressing proliferation, migration and invasion of CRC cells

Epigallocatechin-3-gallate blocks the proliferation and migration of SW620 cells induced by PAR2-AP and factor VIIa via inhibition of the ERK1/2 and NF-κB pathways [137].

Baicalin suppressed migration and invasion of CRC cells by suppressing TLR4/NF-κB signaling pathway [93]. Baicalin triggers apoptosis, inhibits migration, and enhances anti-tumor immunity in colorectal cancer via TLR4/NF-κB signaling pathway [93].

#### Polyphenols targeting PI3K/Akt pathways in suppressing proliferation, migration and invasion of CRC cells

Apigenin is a natural flavonoid, inhibits cell migration, invasion, and metastasis through NEDD9/Src/Akt cascade in colorectal cancer cells [138].

Hydroxysafflor yellow A, is the major active ingredient in traditional Chinese medicine safflower extract, and it is also the water-soluble component with the highest content and the strongest pharmacological effect in safflor yellow [139], suppressed proliferation, migration, invasion, and EMT in HCT116 CRC cells by activating the PPARγ/PTEN/Akt signaling pathways [140].

18 β-glycyrrhetinic acid can effectively inhibit colorectal cancer cell proliferation, invasion and migration probably through suppressing PI3K and STAT3 signaling pathways [141].

#### Polyphenols targeting other signaling pathways in suppressing proliferation, migration and invasion of CRC cells

( −)-Epigallocatechin-3-gallate inhibits Met signaling, proliferation, and invasiveness in human CRC cells [142].

Both quercetin and quercitrin effectively inhibited cell migration and invasion by inducing the MET through the JNK signaling pathway [143].

Luteolin was found to upregulate miR‐384 and downregulate PTN expressions both in CRC cells and tissues. miR‐384 inhibition and PTN overexpression partially reversed the inhibition of HT‐29 cells migration and invasion induced by luteolin [144].

Wogonin suppresses EMT development and the carcinogenic process of CC through the IRF3mediated Hippo signaling pathway [145].

Anti-proliferation and anti-migration effects of epigallocatechin gallate against colorectal-cancer SW480, SW620, and LS411N cells by downregulating the expression of STAT3 [146].

#### Polyphenols with other mechanisms in suppressing proliferation, migration and invasion of CRC cells

Resveratrol significant upregulation of Sirt1 expression, concurrent downregulation of FAK (focal adhesion kinase) activity, and effective inhibition of focal adhesion formation regulates CRC cell invasion [147]. Thus resveratrol suppressed the proliferation and invasion/metastasis of colon cancer cells by activating Tristetraprolin(an AU-rich element-binding protein that regulates mRNA stability and has decreased expression in human cancer) [148].

Rosmarinic acid Consistent inhibition of both activity and expression of matrix metalloproteinase-2 (MMP-2) and -9 (MMP-9) was observed in LS 174-T cells and animal models inhibit migration, adhesion, and invasion of CRC cells [149]. And by inhibiting the non-inflammatory effects of COX-2, Rosmarinic acid could effectively inhibit colon cancer metastasis [150].

Plasma-isolated anthocyanins and their metabolites significantly decrease migration of colon cancer cells in vitro (HT-29 and caco-2) [151].

**Fig. 10:**
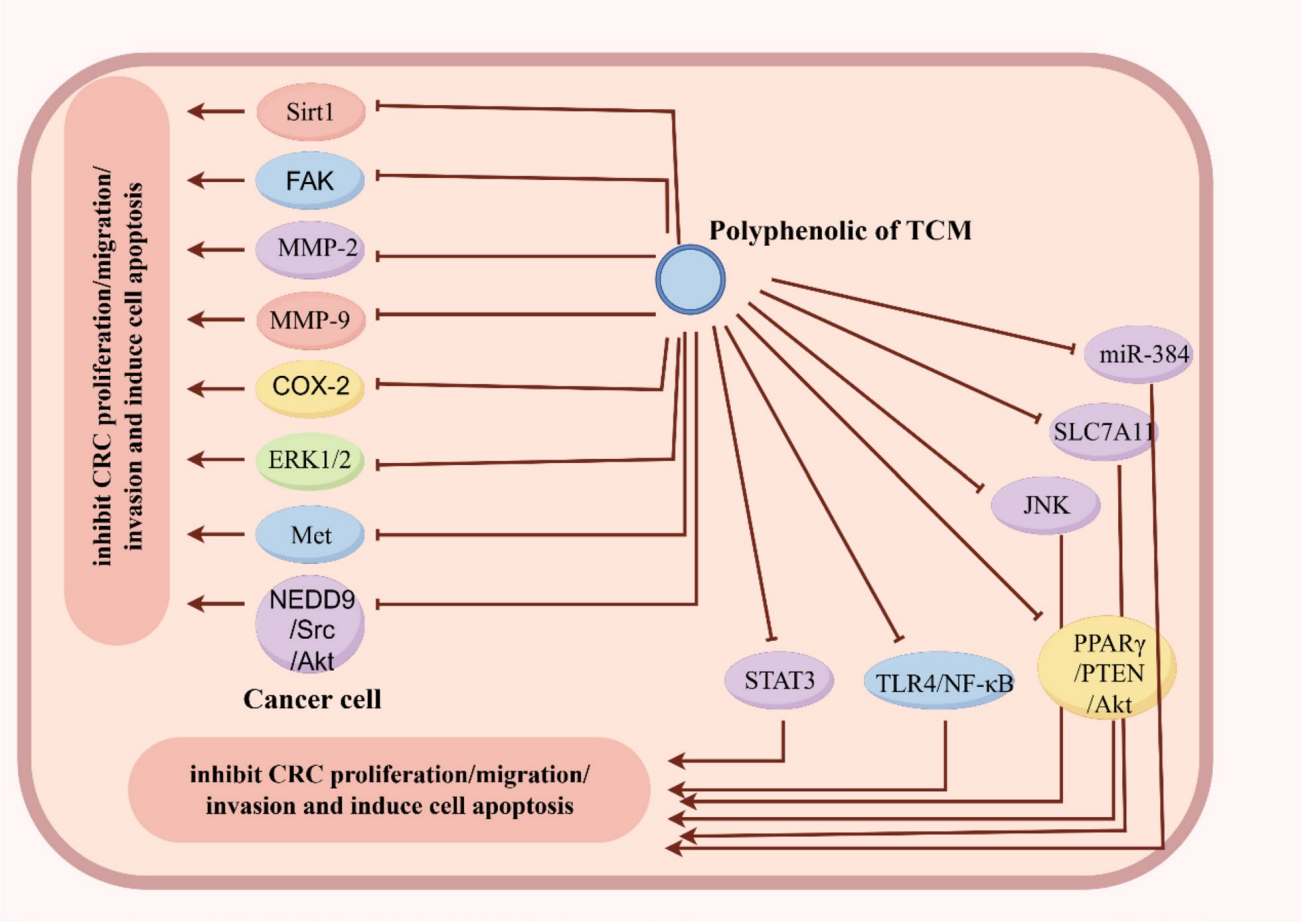
Schematic diagram illustrating the molecular mechanism by which polyphenolic compounds from TCM suppresses colorectal cancer cell proliferation, migration, and invasion. Created in https://www.figdraw.com. DI: TUSIYb4af4

Gingerenone A (GA) significantly inhibits the proliferation of CRC cells both in vitro and in vivo by promoting ferroptosis, thus GA was found to directly interact with SLC7A11, affecting its stability by inducing ubiquitin-mediated degradation of SLC7A11, suppresses SLC7A11 protein levels and induces ferroptosis in CRC cells [152] (Fig. 10).

### Regulating the function of tumor cell mitochondria and controlling the generation of ROS

**Fig. 11:**
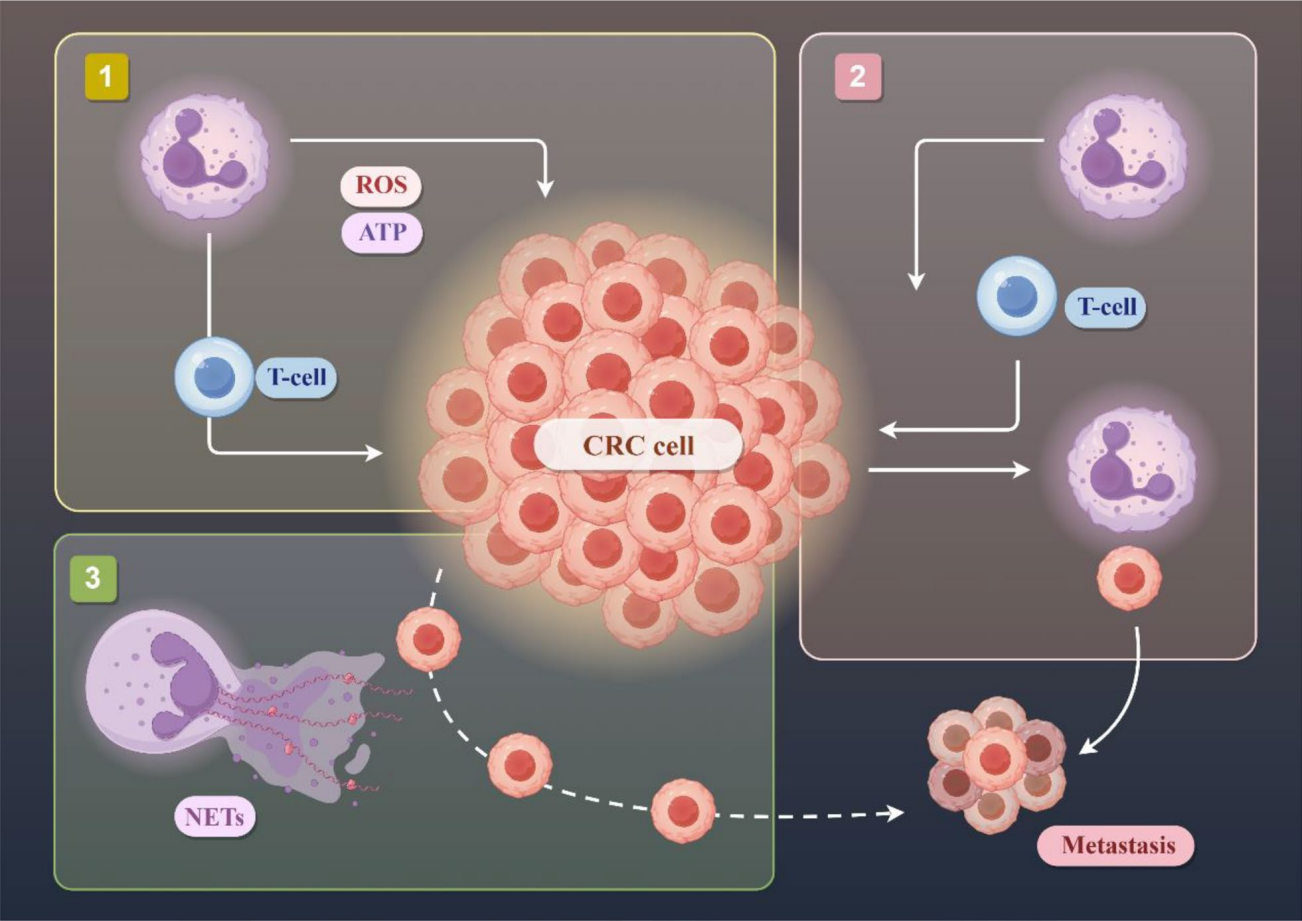
Mechanism diagram of ROS regulation in CRC cell metastasis. Created in https://www.figdraw.com. DI: OIPSAb53ac

Reactive oxygen species (ROS), play a role in various cancer-related processes, including apoptosis, autophagy, necroptosis, ferroptosis, and DNA damage [143, 153–156]. In the context of CRC, ROS are instrumental in gut pathology, targeted therapy, and drug resistance [157, 158] (Fig. 11).

#### Ferroptosis-inducing polyphenols

Combined curcumin and metformin treatment in CRC models, increased ROS production, triggering activation of the NRF2/KEAP1 pathway. This pathway elicits an antioxidant response to manage oxidative stress in CRC cell lines. Notably, the curcumin and metformin promoted lipid peroxidation and downregulated xCT levels, suggesting the induction of ferroptosis. Ferroptosis has been shown to activate autophagy, which helps eliminate cells potentially damaged by the increased oxidative stress. Curcumin and metformin effectively diminished the migratory ability of CRC cells [159].

Emodin could induce ferroptosis in CRC cells by NCOA4-mediated ferritinophagy and inactivation of the NF-κb pathway [160]. However, the exact mechanism by which Emodin regulates the NF-κB pathway in cancer cells is not yet clearly understood, and further studies are required to identify this mechanism.

Erianin attenuated KRAS-driven cancer growth and metastasis in KRASG13D CRC cells and mouse models. Specifically, erianin exerts its effects by interacting with KRASG13D and inducing autophagy-dependent ferroptosis, which suppresses cell proliferation and metastatic phenotypes [161]. This study provides key insights into promising avenues for the treatment of metastatic KRAS mutant CRC and present evidence for the beneficial effects of erianin on KRAS^G13D^ CRC.

Gingerenone A(GA), a unique phenolic component extracted from Zingiber officinale Roscoe, demonstrates a wide range of biological activities, including suppression of proliferation and senescence in breast cancer cells, as well as anti-inflammatory and antiviral effects [162–164]. GA significantly inhibits the proliferation of CRC cells both in vitro and in vivo by promoting ferroptosis. Mechanistically, GA was found to directly interact with SLC7A11, affecting its stability by inducing ubiquitin-mediated degradation of SLC7A11. This process suppresses SLC7A11 protein levels and induces ferroptosis in CRC cells [152].

#### Polyphenols targeting JNK pathways in regulating the function of tumor cell mitochondria and controlling the generation of ROS

Gambogenic acid induced Noxa-mediated apoptosis by activating the ROS/IRE1α/ JNK signaling pathway in CRC both in vitro and in vivo [165]. This study demonstrates that the ROS/ JNK/Noxa axis contribute to Gambogenic acid -induced apoptosis in CRC.

Oblongifolin C (OC) and guttiferone K (GUTK) are two anticancer compounds extracted from Garcinia yunnanensis Hu, the combination of OC and GUTK markedly increased cleavage of casapse-3 and PARP, and enhanced cellular ROS production and increased JNK protein phosphorylation, have apoptosis-inducing effects in HCT116 cells in vitro [166].

Echinatin, a retrochalcone compound is a major component in licorice (Glycyrrhiza species) and has potential pharmacologic activities such as antiinflammatory, antioxidant, anticancer, and antivirus, as well as heart and liver-protective effects [167, 168]. Echinatin increased cell cycle arrest and ROS-mediated apoptosis by inducing the p-JNK and p-p38 in CRC cells [169]. To demonstrate the anticancer effect, a large quantity of Ech would be required for additional in vivo experiments. Compared to other drugs, the isolation and purification of Ech are quite challenging, and a large-scale purification of Ech would allow us to further verify the anticancer effect of Ech from the additional in vivo experiments.

#### Polyphenols targeting PI3K/AKT pathways in regulating the function of tumor cell mitochondria and controlling the generation of ROS

Quercetin inhibited the invasion and migration of advanced metastatic colon cancer LOVO cells under hypoxia through inhibition of ROS and HIF-1α expression and the downregulation of PI3K/AKT pathway [170]. In all, quercetin can compensate for the weak migration inhibition of 5-FU without affecting its efficacy. Combining 5-Fu with quercetin is a potentially effective combination of inhibition of invasion and migration of LOVO cells. But mechanisms for synergies between them needs further studies.

Anticancer activity of Secoisolariciresinol diglucoside in CRC is associated with the induction of GSDMD‐dependent pyroptosis by SDG through the generation of ROS/P13K/AKT/BAK‐mitochondrail apoptosis pathway [171].

#### Polyphenols with other mechanisms in regulating the function of tumor cell mitochondria and controlling the generation of ROS

Quercetrin induced apoptotic cell death in the SW620 cancer cell line was mediated by the excessive generation of ROS, having a potential role in suppressing human colorectal cancer via inhibition of tumor cell proliferation, induction of cell cycle arrest and apoptosis and enhancement of oxidative stress [172]. And quercetin induces apoptosis in HT29 cells by generating ROS through partial inhibition of COX-2 [173]. However, cytotoxicity seen at 77.4 mM Quercetin on HCT15 cells could be attributed to activation of extrinsic apoptotic pathway.

Pharmacological studies have shown that Avenanthramides, which belong to the phenolic alkaloids and are unique to oats, exhibit anti-inflammatory, antioxidative, and anti-cancer properties [174].

Avenanthramide A directly binds to the ATP-binding domain of oncogenic protein DDX3, selectively kills tumor cells in which DDX3 are highly expressed [175].

Carnosic Acid, the main antioxidant compound of Rosmarinus officinalis L., induces anti-proliferative and apoptotic effects in HCT116 cells through interference with STAT3 signaling pathway via generation of ROS [176]. These findings warrant further research to understand the potential of Carnosic Acid as chemopreventive or chemotherapeutics agent against colorectal cancer progress.

Epigallocatechin-3-gallate reduced hemin-induced proliferation and colon carcinogenesis through Nrf2-inhibited mitochondrial ROS accumulation [177]. EGCG pretreatment did not affect Keap1 expression levels which were decreased by hemin treatment. Previous researchers oxidative stress-mediated GSK3β inhibition and PKC activation are critical for Keap1-independent Nrf2 signaling activation. But further study is needed for identification of detailed hemin and EGCG-regulated Nrf2 signaling pathway.

Hemistepsin A (HA) is a sesquiterpene lactone isolated from Hemistepta lyrata Bunge which has been used for the treatment of colon diseases, such as diarrhea and anal fistula [178]. HA suppresses the growth of CRC cells in both in vitro and in vivo models by inhibiting the PDK1 activity, thereby enhancing the metabolic shift from glycolysis to OXPHOS, and consequently inducing mitochondrial ROS-mediated apoptosis. Moreover achieves its inhibitory action on PDK1 by targeting its lipoamide-binding pocket [179]. Although more pharmacological studies and clinical evidence are required, HsA has shown a potential candidate as a novel PDK1 inhibitor for colorectal cancer treatment.

Phloretin, a known dihydrochalcone, the anticancer activity of phloretin can be attributed to its ability of ROS production, depolarization of MMP, G2/M cell cycle arrest and apoptosis. It further deregulates the β-catenin pathway, inhibits growth and causes cell death [180].

ROS and p53 signalings mediate p38 phosphorylation and caspase activation in Kaempferol stimulated apoptosis in CRCs [181].

Salvianolic acid B, a water-soluble phenolic compound, extracted from Salvia miltiorrhiza, reversed MDR in HCT-8/VCR cells, and the effect is associated with increased ROS levels, which may downregulate P-gp expression and promote tumor cell apoptosis [182].

Sanggenon C-induced apoptosis in HT-29 colon cancer cells as a result of the increased ROS generation and activation of the mitochondrial pathway by the inhibition of the iNOS and decreased NO production [171].

## Inhibition of CRC metastasis via non-canonical signaling pathways

### Polyphenols targeting STAT3 pathways

Epigallocatechin‐3‐gallate inhibits the formation of neutrophil extracellular traps and suppresses the migration and invasion of CRC cells by regulating STAT3/CXCL8 pathway [183].

The 5 suppressive effects of acertannin on AOM/DSS-induced colon tumor growth were 6 associated with decreases in the colonic levels of IL-1β, MCP-1, IL-10, and PD-1 via 7 the down-regulated expression of COX-2 and TOX/TOX2 through the inhibition of 8 STAT3 phosphorylation (activation of STAT3) in the tumor microenvironment [184].

### Polyphenols targeting Nrf2/NF-κB pathways

Pterostilbene might be more potent than Resveratrol in mediating the anti-tumorigenic effect by activation of Nrf2 signaling and the induction of its target genes, such as HO-1 and GR, as well as suppression of pro-inflammatory pathways regulated by NF-κB signaling in the initiation stage [185, 186].

### Polyphenols targeting other signaling pathways

The M2 polarization of TAMs was inhibited by curcumin using MACC1 as a target, therefore inhibiting the malignant behavior of colon cancer cells, including proliferation and migration/invasion [187]. Furthermore, curcumin blocked neurotensin-stimulated IL-8 gene induction and protein secretion and, at a low concentration, blocked neurotensin-stimulated CRC cell migration [188].

Apigenin and FdUMP were combined, redox balance was disrupted by depleting anti-oxidant GSH and inducing ROS, CRC cell proliferation arrest and apoptosis could be achieved through DNA damage and energy shortage by GSH downregulation [189].

Resveratrol, as a potent naturally occurring antioxidant, exerted its broad spectrum of antimetastatic effects through its potent inhibition of CRC cell adhesion, migration, invasion, secretion of MMPs, VEGF and MMP-9 gene expression by decreasing the expression and stability of HIF-1a protein in Lovo cells treated with hypoxia and 2,20-dipyridyl [190].

Rosmarinic Acid antagonized ERK1/2 activation in colon HT-29 and breast MCF-7 cancer cells [191, 192].

### Polyphenols with other mechanisms

Litchi procyanidins significantly inhibited the proliferation and metastasis of colon cancer by increasing the number of CD8 + T cells and decreasing the number of macrophages in TME [193].

Five chemical compounds, namely gallic acid, protocatechuic acid, chlorogenic acid, caffeic acid, and isochlorogenic acid A, were identified and quantified in the Patrinia villosa aqueous extract sample. Suppresses the migration and invasion of CRC cells through the regulation of TGF-β R1-smad2/ 3-E-cadherin and FAK-RhoA-cofilin pathways, the modulation of TMErelated immune cells and cytokines, as well as gut microbiota [194].

## Addressing the translational gap—bioavailability, toxicity, and clinical evidence of TCM polyphenols

The therapeutic potential of traditional Chinese medicine (TCM), particularly its polyphenolic constituents, is increasingly recognized for a wide range of diseases, from cancer to renal fibrosis [195, 196]. However, a significant translational hurdle for these promising compounds lies in their inherent physicochemical and pharmacokinetic limitations. As highlighted throughout this review, many TCM polyphenols, such as curcumin, resveratrol, and epigallocatechin-3-gallate, exhibit poor aqueous solubility, chemical instability, and low oral bioavailability, which severely restricts their clinical efficacy and further development [197]. Therefore, a critical discussion of formulation strategies to overcome these barriers is essential for advancing TCM-based therapeutics from the bench to the bedside.

### Formulation strategies to enhance bioavailability

To address the poor biopharmaceutical properties of TCM polyphenols, a diverse array of nano-formulated delivery systems (NDSS) has been extensively explored. These strategies aim to improve solubility, protect the drug from degradation, enhance intestinal permeability, and facilitate targeted delivery.

#### Lipid-based nanocarriers

Lipid-based systems are among the most widely used platforms for improving the oral bioavailability of lipophilic polyphenols. Liposomes, solid lipid nanoparticles (SLNs), and nanostructured lipid carriers (NLCs) can encapsulate hydrophobic compounds, protecting them from the harsh gastrointestinal environment and enhancing their lymphatic transport, thereby bypassing first-pass metabolism. For instance, encapsulating curcumin in liposomes has been shown to significantly increase its stability, with 80% of the initial curcumin remaining after 16 weeks, a stark contrast to its rapid degradation in free form. Similarly, quercetin-loaded SLNs dramatically improved its absorption from 40 to 90%. These carriers not only enhance solubility but can also be engineered for sustained or controlled release, as demonstrated with triptolide-loaded liposomes that prolonged the drug's half-life and reduced its toxicity [197].

#### Polymeric nanoparticles and micelles

Polymeric nanoparticles offer another robust platform. Using biocompatible polymers like PLGA (poly (lactic-co-glycolic acid)) or PEG (Polyethylene Glycol), these systems provide a protective shell for labile compounds, enabling controlled release and targeted delivery. For example, ginkgolide B-loaded polymeric nanoparticles showed an 11-fold increase in maximum plasma concentration and a 3.4-fold longer half-life compared to the free drug [197]. Polymeric micelles, formed by the self-assembly of amphiphilic block copolymers, are particularly effective at solubilizing hydrophobic drugs within their core. A notable example is the co-delivery of quercetin and alantolactone in micelles (QA-M), which not only improved the solubility of both drugs but also synergistically enhanced their anticancer and immunomodulatory effects [197, 198].

#### Inorganic and hybrid nanocarriers

Inorganic nanocarriers like mesoporous silica nanoparticles (MSNs) and gold nanoparticles (GNPs) offer unique advantages, such as high loading capacity and theranostic capabilities. MSNs can co-deliver multiple agents, as seen with the immunomodulator Astragaloside III and the photosensitizer Ce6 for combined cancer therapy. GNPs, with their photothermal properties, can be used for synergistic chemo-photothermal therapy, as demonstrated with oridonin-loaded GNPs for lung cancer. To overcome the limitations of single-component systems, hybrid nanoparticles combine the strengths of different materials. For instance, lipid-coated MSNs were developed for the thermo- and pH-responsive co-delivery of the alkaloids evodiamine and berberine, improving their solubility and reducing side effects [196, 197].

#### Self-assembled natural product nanoparticles

A particularly innovative and "green" strategy involves the carrier-free self-assembly of TCM active components themselves. Driven by non-covalent interactions like hydrogen bonding, π-π stacking, and hydrophobic effects, compounds like berberine can form stable nanoparticles with other molecules such as cinnamic acid, baicalin, or rhein. These self-assembled nanoparticles (SANs) not only enhance the solubility and bioavailability of the individual components but also often exhibit synergistic therapeutic effects, such as enhanced antibacterial or antitumor activity, without the need for synthetic carriers [196, 197, 199]. This approach, inspired by TCM compatibility theory, represents a promising direction for developing simple, safe, and effective nanomedicines.

### Toxicological considerations

While these nanoformulations are designed to enhance efficacy, a thorough evaluation of their safety profile is paramount. The very properties that improve bioavailability, such as altered tissue distribution and enhanced cellular uptake, could also lead to unintended toxicity. The safety of the nanocarrier materials themselves must be considered. While lipids and many polymers like PLGA and PEG are generally regarded as safe, the long-term in vivo fate of other components, such as inorganic materials or novel surface coatings, requires rigorous investigation. Furthermore, the enhanced permeability and retention (EPR) effect, while enabling tumor targeting, is not universally efficient, and concerns about off-target accumulation and potential chronic toxicity of non-biodegradable nanomaterials remain. For example, despite its potent anti-inflammatory effects, the clinical use of triptolide is severely limited by its toxicity. However, encapsulating it in nanostructured lipid carriers (NLCs) or other delivery systems has been shown to significantly mitigate its hepatotoxicity and nephrotoxicity while maintaining its therapeutic effect [198]. This highlights the crucial role of formulation in not only improving efficacy but also in enhancing the therapeutic window of potent, yet toxic, natural products.

### Clinical trial evidence

The ultimate test of these formulation strategies lies in clinical translation. Although many studies remain preclinical, the clinical application of TCM polysaccharides, such as Astragalus polysaccharides (APS) and Lentinan (LNT), as adjuncts in cancer therapy is well-documented. For instance, clinical studies have shown that Astragalus polysaccharide injection (PG2) can normalize the neutrophil-to-lymphocyte ratio in lung cancer patients undergoing immunotherapy, improve quality of life, and reduce chemotherapy-induced toxicity. Similarly, Lentinan has been shown to enhance immune function and improve survival in patients with gastric and esophageal cancers when used in combination with chemotherapy [195, 198]. These successes with TCM polysaccharides, which themselves face bioavailability challenges, underscore the potential of advanced delivery systems for other small-molecule TCM components.

However, clinical trials specifically evaluating nanoformulated TCM polyphenols are still scarce. Most current evidence is based on preclinical animal models. The progression of berberine into phase IV clinical studies for other indications offers a promising avenue, and its incorporation into advanced delivery systems could further unlock its potential for diseases like renal fibrosis [195, 196]. The challenge lies in navigating the regulatory pathway for these complex multi-component systems and demonstrating clear clinical superiority over conventional formulations. The future of TCM in modern medicine hinges on high-quality clinical trials that validate not only the efficacy but also the safety of these novel nano-enabled formulations.

### Reconciling TCM complexity with modern drug development

A fundamental challenge in modernizing TCM is reconciling its "multi-component, multi-target" philosophy with the reductionist approach of modern drug development, which often seeks a single, pure compound for a specific target. Nanoformulations offer a potential bridge. They can be designed to deliver defined ratios of multiple active components, mimicking the synergistic effects of a TCM formula while ensuring batch-to-batch consistency and quality control. The self-assembled nanoparticles derived from TCM compound pairs, such as berberine and baicalin, are a perfect example of how modern technology can be used to validate and enhance ancient therapeutic principles [196, 199]. By standardizing the production of these complex nano-assemblies and rigorously investigating their pharmacokinetics and pharmacodynamics, we can begin to deconvolute their mechanisms of action while preserving their inherent multi-targeted nature. This approach, supported by robust preclinical data and well-designed clinical trials, holds the key to successfully translating the therapeutic wisdom of TCM into evidence-based modern medicines.

## Discussion and future perspectives

Tumor metastasis is an important marker of tumor deterioration. A large number of studies have shown that the five-year survival rate of tumors after metastasis will be severely reduced. EMT and tumor angiogenesis play important roles in tumor metastasis. The tumor first undergoes angiogenesis to accumulate energy in the primary location, and then undergoes the EMT process from epithelial cells to mesenchymal cells. After the tumor cells are transformed into mesenchymal cells, they escape the recognition of the immune system and leave the original implantation site to enter the blood vessels or lymph tube, entering into other metastases as the blood circulates. Corresponding protein expression in tumor cells during angiogenesis and EMT will change accordingly, such as the proteins of Vimentin, N-Cad, E-Cad and vascular endothelial growth factor (VEGF) etc. Changes that affect the expression of such proteins can also affect the process of tumor cell metastasis. Mitochondria play an important role in the process of tumor cell metastasis. Mitochondria can provide energy for cell metastasis in the cell, while inhibiting mitochondrial function can inhibit tumor metastasis. This article mostly focuses on autophagy and apoptosis in tumor cells during metastasis, EMT of cancer cells and tumor angiogenesis. In addition, the tumor microenvironment also has a great impact on the tumor. In the tumor microenvironment, M1 type macrophages are transformed into M2 type macrophages under the influence of tumor cells to promote tumor growth. Tumor cells express PD-L1 and bind to PD1 on the surface of immune system T cells, so that T cells recognize tumor cells as normal cells, and immune escape occurs.

As a treasure of China, TCM is characterized by low toxicity and minimal side effects., less damage to the body with multiple targets. TCM prescription has wonderful effects on the treatment of tumors, but its mechanism for treating cancer is difficult to elucidate. Many TCM prescriptions have been clarified to have good effect on anticancer treatment clinically such as Fuzheng Kang-Ai decoction, Jinfu’an Decoction and YangZheng XiaoJi used for treating lung cancer, Jiedu Recipe for hepatic carcinoma, Jianpi Jiedu decoction and Jiedu Sangen Decoction for colorectal cancer. The clarification of the effective ingredients in the prescription and the specific molecular targeting mechanism of each ingredient require a complex process. In the past ten years, the research on the anti-cancer mechanism of TCM prescription is mostly manifested in the inhibitory effect on the proliferation, migration and invasion of tumor cells during tumorigenesis. But no clarification of its effective ingredients and the anti-cancer molecular mechanism of its ingredients have been elucidated. TCM prescription has many active ingredients, and every ingredient has the characteristics of multiple targets in the body, so to clarify its specific mechanism of action on cancer requires a lot of work. The representative component of Compound Kushen Injection in Chinese patent medicine has multiple targets predicted by network pharmacology, including inhibition of tumor proliferation, invasion, migration, angiogenesis, etc. and Compound Kushen Injection can be used for a variety of cancers including colorectal cancer, breast cancer and liver cancer [200, 201]. Research of the anticancer effect of Polyphenolic in TCM has made considerable progress in recent years, of which are representatives like Apigenin, Resveratrol, and Curcumin [202–205]. Among the monomer compounds in TCM, Resveratrol, Onionin A, Genistein, Curcumin, Magnolol and Honokiol also have related studies in tumor microenvironment and immune regulation [206–212]. For combination with clinical chemotherapy drugs, TCM has function in many ways like prolonging the patient survival time, reducing the dose of chemotherapy drugs, reversing drug resistence, alleviating patient adverse reaction and so on.

This article mostly focuses on the research on Regulation of Polyphenolic Active Substances in TCM on the Metastasis of CRC in the past ten years. The combined application of TCM and clinical medicine can often achieve unexpected results. The combined application of TCM with clinical chemotherapy drugs can enhance the efficacy of clinical chemotherapy drugs, reduce their side effects, and improve the patient’s survival time and quality of life. Polyphenolic Active Substances in TCM are often not unilateral for the treatment of cancer. The inhibitory effect of TCM on metastasis often manifests in inhibiting the expression of EMT-related proteins, thereby inhibiting EMT, inhibiting the expression of angiogenesis-related proteins, inhibiting angiogenesis, regulating cell autophagy, regulating apoptosis, and regulating mitochondrial function. TCM plays an important role in the treatment of cancer. Compared with the progress of clinical chemotherapeutics, the research progress of TCM is slower, and the research on the mechanism of Chinese medicine compound on cancer is still rare. Thereby providing insights and a scientific foundation for future research focusing on the regulatory mechanisms of specific active components in TCM (such as polyphenols) on cancer metastasis, and for the clinical application of TCM or even single-agent active constituents from TCM in treating or adjunctively treating cancer metastasis to achieve better therapeutic outcomes.

## Data Availability

All data generated or analyzed during this study are available in this published article and its Additional files.
